# *Rubus urticifolius* Compounds with Antioxidant Activity, and Inhibition Potential against Tyrosinase, Melanin, Hyaluronidase, Elastase, and Collagenase

**DOI:** 10.3390/ph17070937

**Published:** 2024-07-13

**Authors:** Luis Apaza Ticona, Javier Sánchez Sánchez-Corral, Carolina Díaz-Guerra Martín, Sara Calderón Jiménez, Alejandra López González, Cristina Thiebaut Estrada

**Affiliations:** 1 Organic Chemistry Unit, Department of Chemistry in Pharmaceutical Sciences, Faculty of Pharmacy, University Complutense of Madrid, Plza. Ramón y Cajal s/n, 28040 Madrid, Spain; 2 Department of Organic Chemistry, Faculty of Sciences, University Autónoma of Madrid, Cantoblanco, 28049 Madrid, Spain; 3 COBIOSA, Road of Alpedrete, 6, 28400 Collado Villalba, Spain; cristina.thiebaut@cobiosa.com

**Keywords:** *Rubus*, fruit, anti-hyperpigmentation, melanin, anti-inflammatory, antioxidant, anti-aging

## Abstract

In our study, using chromatographic techniques, we isolated three bioactive compounds, which were structurally elucidated as (*E*)-2-(3-(3,4-dimethoxyphenyl)acrylamido)-*N*-methylbenzamide (**1**), 4-Hydroxyquinoline-2-carboxylic acid (**2**), and (*E*)-2-Cyano-3-(4-hydroxyphenyl)acrylic acid (**3**), using spectroscopic methods. The anti-melanogenic, anti-inflammatory, antioxidant, and anti-aging properties were evaluated in vitro by measuring the activity of pharmacological targets including tyrosinase, melanin, NF-κB, hyaluronidase, elastase, collagenase, and Nrf2. Our results show that compound **1** is the most active with IC_50_ values of 14.19 μM (tyrosinase inhibition), 22.24 μM (melanin inhibition), 9.82–12.72 μM (NF-κB inhibition), 79.71 μM (hyaluronidase inhibition), 80.13 μM (elastase inhibition), 76.59 μM (collagenase inhibition), and 116–385 nM (Nrf2 activation) in the THP-1, HEK001, WS1, and HMCB cells. These findings underscore the promising profiles of the aqueous extract of *R. urticifolius* at safe cytotoxic concentrations. Additionally, we report, for the first time, the isolation and characterisation of these nitrogenous compounds in the *R. urticifolius* species. Finally, compound **1**, isolated from *R. urticifolius*, is a promising candidate for the development of more effective and safer compounds for diseases related to skin pigmentation, protection against inflammation, and oxidative stress.

## 1. Introduction

Hyperpigmentation is a complex process involving a series of molecular and cellular mechanisms that can be altered by various pathological conditions. Under normal conditions, melanin, the pigment responsible for skin colour, is produced from tyrosine through a series of enzymatic reactions [1]. The key enzyme in this synthesis is tyrosinase, which converts tyrosine to L-3,4-dihydroxyphenylalanine (L-DOPA), a crucial step in melanin production [2]. However, in the context of hyperpigmentation, this process can be altered due to the overexpression or hyperactivation of tyrosinase, resulting in increased melanin production and thus excessive skin pigmentation. This change may be influenced by various factors such as sun exposure or inflammation [3].

Inflammation plays a crucial role in the development and perpetuation of hyperpigmentation. One of the main inflammatory mediators involved is the nuclear factor kappa-light-chain-enhancer of activated B cells (NF-κB), a transcription factor that regulates the expression of genes related to inflammation and the immune response [4,5]. NF-κB activation during inflammation can directly or indirectly stimulate tyrosinase production and other factors that promote hyperpigmentation, highlighting the close relationship between inflammation and skin pigmentation [6,7].

Furthermore, oxidative stress, characterised by an imbalance between the production of reactive oxygen species (ROS) and the antioxidant capacity of the skin, can significantly contribute to hyperpigmentation [8]. ROS can both trigger NF-κB activation and other inflammatory signalling pathways as well as directly affect the activity of tyrosinase and other enzymes involved in melanin synthesis, thereby altering melanin production and contributing to hyperpigmentation [9]. To counteract oxidative stress, skin cells rely on antioxidant systems such as nuclear factor erythroid 2-related factor 2 (Nrf2), a transcription factor that regulates the expression of genes involved in antioxidant defence [10]. Nrf2 activation can protect the skin from oxidative damage by increasing the expression of antioxidant enzymes such as superoxide dismutase and catalase [11].

Moreover, the generated oxidative stress leads to an increase in the production of enzymes such as hyaluronidase, collagenase, and elastase, which can degrade the structural components of the skin, influencing hyperpigmentation [12]. In the case of hyaluronidase, it degrades hyaluronic acid, preventing it from performing its protective, reparative, healing, and hydrating functions [13]. Additionally, hyaluronidase activates membrane receptors that trigger pathways converging in NF-κB activation [14]. On the other hand, elastase degrades elastin, preventing it from fulfilling its function of providing elasticity to the skin. Likewise, elastase activates NF-κB translocation to the nucleus to carry out its functions [15]. Finally, collagenase degrades collagen, a protein that provides structure and support to the skin [16].

In contrast to conventional medications, natural products have emerged as promising alternatives to treat skin hyperpigmentation, offering gentler options that are less likely to cause unwanted side effects [17]. Numerous botanical species have been studied for their depigmenting and anti-inflammatory properties, providing a wide range of options for those seeking more natural treatments. Among these species are plants belonging to the genus Hypericum, as well as *Aloe vera*, *Azadirachta indica*, *Cyperus rotundus*, *Embica officinalis*, *Glycyrrhiza glabra*, *Holarrhena antidysentrica*, *Verbena officinalis*, *Magnolia officinalis*, *Melissa officinalis*, *Panax ginseng*, and *Syzygium aromaticum* [18]. Rubus is one of the most studied genera for this purpose.

The Rubus genus comprises around 1700 accepted species from the Rosaceae family, widely distributed worldwide, especially in temperate regions [19]. Rubus species have been used since ancient times to heal wounds, insect bites, boils, burns, inflammation, and pain [20,21]. Recent studies have shown that fruits of the Rubus genus have antioxidant, anti-inflammatory, and anti-aging properties, regulating different enzymes in the body and acting as good scavengers of free radicals [21]. Currently, the number of compounds isolated from species of this genus amounts to 260 [21], including phenolic, terpenic, and alkaloid compounds [22,23,24,25].

In this study, we investigated *Rubus urticifolius* Poir. (*R. urticifolius*), a woody shrub belonging to the Rubus genus, whose fruits are commonly known as blackberries. It is also known in endemic areas as “Khari-Khari” and “Mora de monte” [26]. This species is native to various areas of Central and South America, including Colombia, Brazil, and Bolivia [27]. Its traditional use lies in its action against inflammation caused by bruises [28]. Both the leaves and the fruit are used, either in the form of an infusion or poultice or by directly consuming the blackberries [29]. The infusion is also used as an eye wash for erysipelas and ocular inflammation, as well as to relieve the burning and itching sensations caused by skin conditions. Additionally, gargling with boiled leaves and flowers can reduce throat inflammation [30].

Previous research has shown that most species belonging to the Rubus genus have antioxidant, anti-aging, and anti-inflammatory properties. For example, studies by Jung et al. [31] have shown that methanolic extracts of the berries of *R. fruticosus* and *R. coreanus* inhibit cyclooxygenase-2 (COX-2) expression at a concentration of 20 μg/mL in inflamed intestinal epithelial cells Caco-2. In another case, Ding [32] demonstrated that ethanolic, ethyl acetate, and *n*-butanol extracts of *R. chingii* eliminate 2,2-diphenyl-1-picrylhydrazyl (DPPH)-type free radicals, with EC_50_ values of 17.9, 3.4, and 4.0 μg/mL, respectively. Additionally, as evidenced by Krauze-Baranowska et al. [33], the phenolic extract of *R. idaeus* buds has strong antioxidant activity (EC_50_ = 19.4 μg/mL). Similarly, Argoti et al. [34] showed that the ethanolic extract of the *R. urticifolius* species has 81.62% (green fruit) and 72.03% (ripe fruit) antioxidant activity, expressed as inhibition percentages of linoleic acid oxidation (*β*-carotene bleaching method).

In this context, the current article shows how nitrogenous compounds have been isolated and analyses their composition. This is followed by an analysis of their anti-inflammatory, antioxidant, and anti-aging properties, as well as their role against hyperpigmentation, based on their inhibitory action against targets regulating these processes such as tyrosinase, melanin, hyaluronidase, elastase, and collagenase.

## 2. Results

### 2.1. Isolation and Characterisation of Compounds of R. urticifolius

Upon analysing the UV spectra of the *n*-hexane extract of *R. urticifolius* (HERu) and dichloromethane/methanol extract of *R. urticifolius* (DMCERu), we observed a slight band in the intermediate region (261–332 nm), indicating the presence of unsaturated compounds (double bonds). The maximum absorption for HERu was 276 nm, and for DMCERu, it was 261 nm (Figure S1).

In the IR spectrum of DMCERu, signals corresponding to organic compounds were identified. The band at 3200 cm^−1^ indicated an O-H/N-H stretch, possibly from phenols, amides, or amines. The band at 2900 cm^−1^ corresponded to a typical O-H stretch of a carboxylic acid. Additionally, a characteristic carbonyl band at 1750 cm^−1^ suggested the presence of acids, amides, or esters. A band at 1600 cm^−1^ indicated a C=C stretch of alkenes while another at 1500 cm^−1^ suggested a C-C stretch from aromatic systems and olefinic signals (Figure S2).

To determine the presence of natural metabolites and the complexity of each sample, ^1^H NMR spectroscopy was employed (Figures S3–S7). The spectrum of the aqueous extract of *R. urticifolius* (AqERu) showed aromatic signals around *δ*_H_ 6.5–7.0 ppm, potentially suggesting the presence of compounds containing aromatic rings such as polyphenols, flavonoids, or alkaloids. Signals between *δ*_H_ 3.2–4.5 ppm could correspond to protons near alcohols, ethers, or ketones, such as glycosylated natural products (Figures S3, S6 and S7).

In the ^1^H NMR spectrum of HERu (Figure S4), a significant number of aliphatic signals were observed (between 0.8–2.2 ppm), which likely correspond to essential oils and predominantly hydrocarbon compounds with limited functional groups. In the ^1^H NMR spectrum of DCMERu (Figure S5), a higher number of allylic signals (5.0–5.7 ppm) was observed, suggesting the presence of more complex chemical structures with potential biological activity.

Finally, the ^1^H NMR spectra of both HERu and DMCERu exhibited similar fingerprints, suggesting the presence of aromatic compounds. The signals around *δ*_H_ 7.0 ppm and *δ*_H_ 5.5 ppm could evidence the existence of aromatic and vinyl hydrogens, respectively. In a more shielded area, signals at *δ*_H_ 3.8 ppm may correspond to hydrogens neighbouring a heteroatom such as N-CH_3_ or O-CH_3_. Aliphatic signals typical of simple chain hydrocarbons or essential oils were observed around 2.5–0.9 ppm (Figures S6 and S7).

Based on these results and those obtained from biological assays, DCMERu was fractionated, leading to the isolation and characterisation of three compounds using NMR, IR, and MS techniques.

The ^1^H NMR spectra of compound **1** revealed a broad singlet at 8.75 ppm, corresponding to the H-6′ hydrogen. The signal for H-2′ (a doublet of doublets) at 7.74 ppm was coupled with the H atoms of the aromatic ring H-5′ and H-6′. Additionally, a correlation between H-2 (7.54 ppm) and H-3 (6.76 ppm) was noticed since both signals exhibited a coupling constant of 15.4 Hz, which is characteristic of olefinic signals (Figure S8).

**Figure 1:**
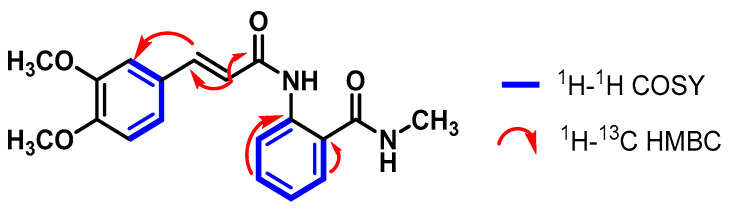
Correlations established in compound **1**.

In the aliphatic region of the spectrum, two singlets were observed at 3.85 ppm and 3.80 ppm, corresponding to the three methoxy hydrogens at positions C-3′ and C-4′. Furthermore, a doublet at 2.83 ppm with a coupling of 3.8 Hz was observed, corresponding to three methyl hydrogens of an amino group (Figure S9). Although the methoxy (-OMe) and methyl (-NMe) hydrogens had direct correlations with their respective carbons in the ^1^H-^13^C HSQC and DEPT-135 spectra, they are not specified here due to the lack of specific proton-to-carbon assignment in those functional groups. With the ^1^H-^13^C HMBC spectrum, a correlation between the H-3 atom (6.76 ppm) and the COOH carbons (*δ*_C_ 166.7 ppm) and C-4 (*δ*_C_ 162.2 ppm) was established, showing the presence of a carboxylic acid at C-4 and a carbonyl at C-3 (Figure 1).

Two-dimensional NMR was performed on compounds **2** and **3** for their final characterisation as both have the same molecular weight and similar structures. Additionally, infrared spectroscopy was used to determine the presence or absence of the stretching band of nitrile functional groups (2200–2400 cm^−1^).

The IR spectrum of compound **2** showed signals at 1000 cm^−1^ (O-H curve) and 1100 cm^−1^ (C-N stretch); however, no band was observed in the specified range for the nitrile group (Figure S10). In contrast, the IR spectrum of compound **3** showed signals at 800 cm^−1^ (N-H wag), 1200 cm^−1^ (C-O stretch), 1500 cm^−1^ (C-C stretch), 1600 cm^−1^ (C=C stretch), and 3300 cm^−1^ (N-H stretch). Unlike the IR spectrum of compound **2**, the IR spectrum of compound **3** showed a band at 2230 cm^−1^, corresponding to the C≡N stretch (Figure S15).

In the ^1^H NMR spectrum of compound **2**, a signal group consisting of two pairs of doublets at 8.28 ppm and 7.89 ppm, corresponding to the hydrogen pairs H-5 and H-8, respectively, was observed. Additionally, in the ^1^H-^1^H COSY spectrum, coupling between H-5 hydrogen and the corresponding double triplets with H-6 (7.78 ppm) and H-7 (7.48 ppm) hydrogen was noticed while a less shielded singlet was observed at 7.02 ppm, corresponding to the H-3 atom (Figures S11–S13).

**Figure 2:**
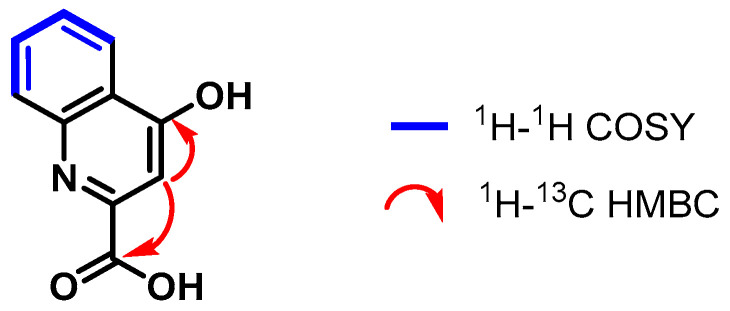
Correlations established in compound **2**.

The ^1^H-^13^C HSQC and DEPT-135 spectra helped assign the carbons C-6 (*δ*_C_ 134.2 ppm), C-5 (*δ*_C_ 126.0 ppm), C-7 (*δ*_C_ 125.8 ppm), C-8 (*δ*_C_ 120.4 ppm), and C-3 (*δ*_C_ 110.2 ppm) that correspond to the proton signals explained above. Moreover, through the analysis of the ^1^H-^13^C HMBC spectrum, the ^2.3^*J*_CH_ correlations were established between the H-3 atom (7.02 ppm) and the COOH (*δ*_C_ 166.7 ppm) and C-4 (*δ*_C_ 162.2 ppm) carbons (Figure 2).

In the ^1^H NMR spectrum of compound **3**, a signal group consisting of two pairs of doublets at 7.94–7.92 ppm and 6.91–6.89 ppm, corresponding to the hydrogen pairs H-2′/H-6′ and H-3′/H-5′, was observed. This pattern change is typical of benzyl systems substituted in the *para*-position of the ring [35]. Additionally, the coupling between the H-3′/H-5′ and H-2′/H-6′ atoms of the aromatic system could be observed in the ^1^H-^1^H COSY spectrum. A coupling between the H-2′/H-6′ atoms and the H-3 atom was also evidenced (Figures S16–S18).

**Figure 3:**
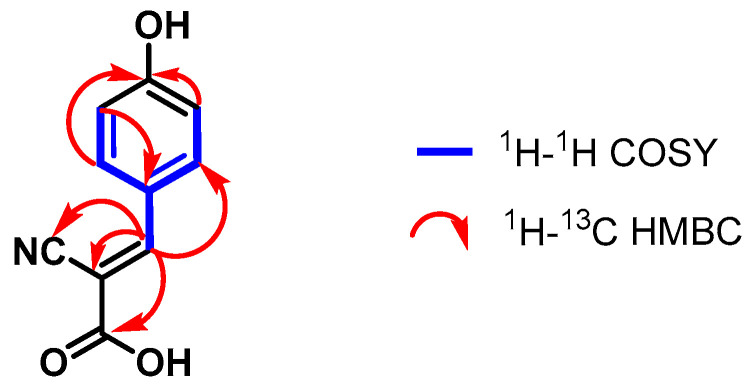
Correlations established in compound **3**.

The ^1^H-^13^C HSQC and DEPT-135 spectra helped assign the carbons C-5 (*δ*_C_ 155.8 ppm), C-2′/C-6′ (*δ*_C_ 134.9 ppm), and C-3′/C-5′ (*δ*_C_ 117.2 ppm) that correspond to the proton signals explained above. Moreover, through the analysis of the ^1^H-^13^C HMBC spectrum, the ^2.3^*J*_CH_ correlations were established between the carbons C-1 (*δ*_C_ 165.9 ppm), C-4′ (*δ*_C_ 164.0 ppm), C-1′ (*δ*_C_ 124.5 ppm), and C-2 (*δ*_C_ 99.5 ppm) and their respective hydrogens (Figure 3).

**Figure 4:**
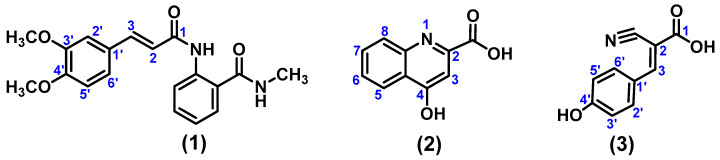
(*E*)-2-(3-(3,4-dimethoxyphenyl)acrylamido)-*N*-methylbenzamide (**1**), 4-Hydroxyquinoline-2-carboxylic acid (**2**), and (*E*)-2-Cyano-3-(4-hydroxyphenyl)acrylic acid (**3**) isolated from the dichloromethane/methanol extract of *R. urticifolius*.

Once the compounds were characterised and analysed, they were identified as (*E*)-2-(3-(3,4-dimethoxyphenyl)acrylamido)-*N*-methylbenzamide (**1**), 4-hydroxyquinoline-2-carboxylic acid (**2**), and (*E*)-2-cyano-3-(4-hydroxyphenyl)acrylic acid (**3**) (Figure 4).

### 2.2. Viability Assay of the Extracts and Compounds of R. urticifolius

**Figure 5:**
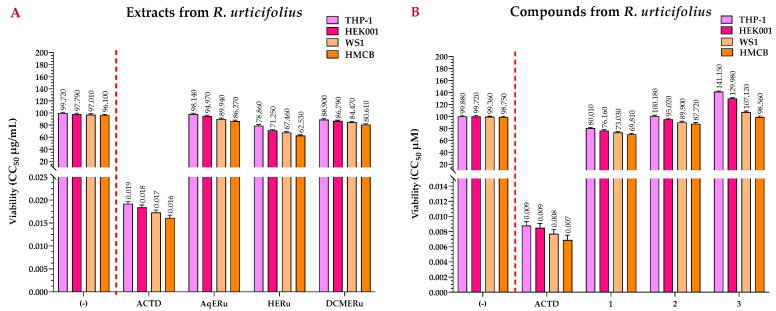
CC_50s_ of the 3-amino-7-dimethylamino-2-methylphenazine hydrochloride salt reagent (NR) (viability) assays calculated for the extracts (**A**) and compounds (**B**) of *R. urticifolius* at 72 h. (-) = Untreated cells (negative control); ACTD = positive control; AqERu = aqueous extract of *R. urticifolius*; HERu = *n*-hexane extract of *R. urticifolius*; DCMERu = dichloromethane/methanol extract of *R. urticifolius*. ^a^ Viability CC_50_ values are the means of three independent assays.

Figure 5A shows the viability values of the extracts from *R. urticifolius*, which are higher than that of Actinomycin D (ACTD). ACTD had CC_50_ values of 0.0184, 0.0173, and 0.0161 μg/mL for the HEK001, WS1, and HMCB cell lines, respectively, and 0.0192 μg/mL for the THP-1 control cell line.

AqERu exhibited CC_50_ values of 94.97, 89.94, and 86.27 μg/mL while DCMERu displayed CC_50_ values of 86.79, 84.47, and 80.61 μg/mL, respectively. Neither exhibited significant higher cytotoxicity than ACTD in the HEK001, WS1, and HMCB cell lines. Regarding their cytotoxicity in the THP-1 control cell line, AqERu and DCMERu had CC_50_ values of 98.14 and 88.90 μg/mL, respectively, showing no cytotoxic effects. In contrast, HERu exhibited slightly higher cytotoxicity than AqERu and DCMERu in the HEK001, WS1, and HMCB cell lines, with CC_50_ values of 71.25, 67.46, and 62.53 μg/mL, respectively. Furthermore, HERu also demonstrated slight cytotoxicity against the THP-1 control cell line, with a CC_50_ value of 78.86 μg/mL (Figure 5A).

None of the three compounds were as cytotoxic as ACTD, with CC_50_ values of 76.16, 95.02, and >100 μM for HEK001; 73.03, 89.90, and >100 μM for WS1; and 69.81, 87.72, and 98.56 μM for HMCB. However, when comparing the compounds with each other, compound **1** showed slightly higher cytotoxicity in both cell lines, followed by compounds **2** and **3**. In the case of the control cell line (THP-1), all the compounds were less cytotoxic than the ACTD (CC_50_ = 0.0069–0.0088 μM), with CC_50_ values of 80.01, >100, and >100 μM, respectively (Figure 5B).

### 2.3. Effects of the Extracts and Compounds of R. urticifolius on the Inhibition of Tyrosinase and Melanin Production

**Figure 6:**
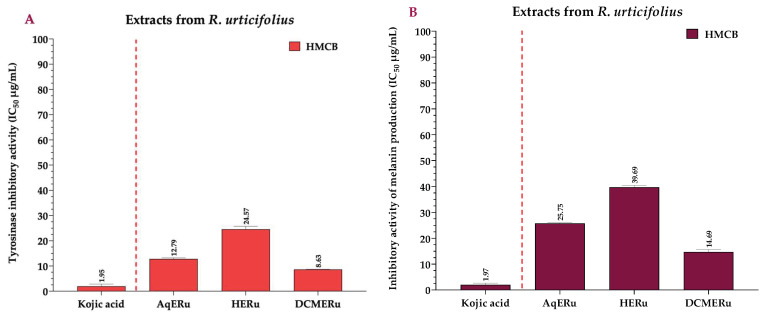
IC_50_ of tyrosinase-inhibitory activity using L-DOPA as substrate, calculated for the extracts (**A**), and IC_50_ of inhibitory activity of melanin production, calculated for the extracts (**B**) at 12 h. Kojic acid = positive control; AqERu = aqueous extract of *R. urticifolius*; HERu = *n*-hexane extract of *R. urticifolius*; DCMERu = dichloromethane/methanol extract of *R. urticifolius*.

DCMERu significantly decreased tyrosinase activity in HMCB cells in a concentration-dependent manner, with an IC_50_ of 8.63 µg/mL. Consequently, the DCMERu exhibited a tyrosinase-inhibitory potential similar to that Kojic acid (*p* < 0.001), which had an IC_50_ of 1.97 µg/mL. Additionally, AqERu (IC_50_ = 12.79 µg/mL) and HERu (IC_50_ = 24.57 µg/mL) had higher IC_50_ values than the Kojic acid (Figure 6A). The assessment of melanin production inhibition potential in the HMCB cell line showed that AqERu, HERu, and DCMERu had IC_50_ values of 25.75, 39.69, and 14.69 µg/mL, respectively. Although the DCMERu displayed higher inhibitory activity than the other extracts, its capacity to inhibit melanin production was lower than that of the Kojic acid (IC_50_ = 1.97 µg/mL) (Figure 6B).

**Figure 7:**
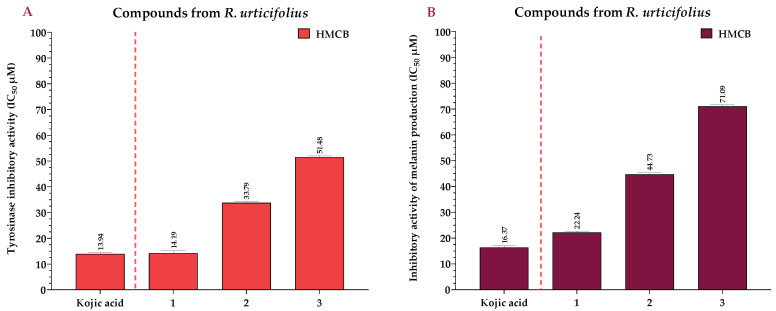
IC_50_ of tyrosinase-inhibitory activity using L-DOPA as substrate, calculated for the compounds (**A**), and IC_50_ of inhibitory activity of melanin production, calculated for the compounds (**B**) at 12 h. Kojic acid = positive control.

Compounds **1–3** exhibited significant reductions in tyrosinase activity in the HMCB cells in a concentration-dependent manner, with IC_50_ values of 14.19, 33.79, and 51.48 µM, respectively. Remarkably, compound **1** exhibited a tyrosinase-inhibitory potential similar to that of the Kojic acid (*p* < 0.001), which had an IC_50_ of 13.94 μM (Figure 7A). Regarding compounds **1**, **2**, and **3**, they inhibited melanin production in the HMCB cell line with IC_50_ values of 22.24, 44.73, and 71.09 µM, respectively. Compound **1** had a higher inhibitory activity than the other compounds, similar to that of the Kojic acid (IC_50_ = 16.37 µM) (Figure 7B).

### 2.4. Anti-Inflammatory Activity of the Extracts and Compounds of R. urticifolius

In Table 1, the IC_50_ values of the extracts for NF-κB inhibition (stimulated with Lipopolysaccharide (LPS)) can be compared to those of Celastrol, which had IC_50_ values of 3.35 µg/mL (THP-1 cells), 3.33 µg/mL (HEK001), 3.29 µg/mL (WS1), and 3.27 µg/mL (HMCB cells). After analysing the results, we can conclude that DCMERu exhibited higher anti-inflammatory activity than AqERu and HERu, as shown by its IC_50_ values of 29.10 µg/mL (THP-1 cells), 28.06 µg/mL (HEK001 cells), 23.03 µg/mL (WS1 cells), and 19.86 µg/mL (HMCB cells).

In Table 1, the IC_50_ values for NF-κB inhibition (stimulated with LPS) of the compounds can be compared to those of the Celastrol, with IC_50_ values of 7.62, 7.57, 7.41, and 7.36 µM in the THP-1, HEK001, WS1, and HMCB cell lines, respectively. Upon analysing the results on the anti-inflammatory activity of the compounds, it was observed that compound **1** exhibited anti-inflammatory activity similar to that of the Celastrol, with an IC_50_ value of 9.82 µM in the HMCB cell line.

### 2.5. Antioxidant Capacity of the Extracts and Compounds of R. urticifolius

**Figure 8:**
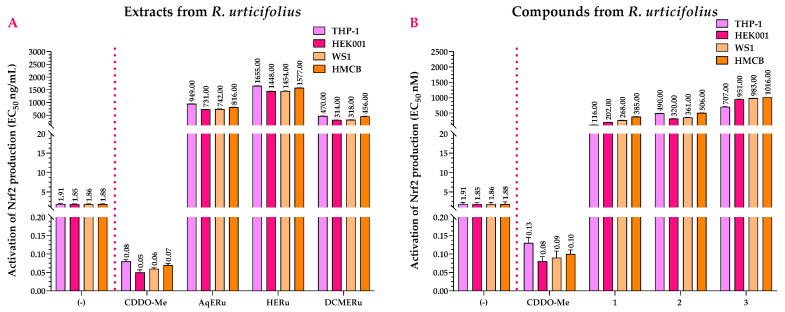
Effects of *R. urticifolius* extracts (**A**) and compounds (**B**) on Nrf2 activation at 72 h. (-) = Untreated cells = negative control; CDDO-Me = positive control; AqERu = aqueous extract of *R. urticifolius*; HERu = *n*-hexane extract of *R. urticifolius*; DCMERu = dichloromethane/methanol extract of *R. urticifolius*. Nrf2 EC_50_ values represent the means of three independent assays.

In the Nrf2 activation assay, AqERu (EC_50_ = 731 and 949 ng/mL) and DCMERu (EC_50_ = 314 and 470 ng/mL) exhibited more pronounced activity in the THP-1, HEK001, WS1, and HMCB cell lines than HERu. However, they did not surpass the activity of 2-Cyano-3,12-dioxo-oleana-1,9(11)-dien-28-oic acid methyl ester (CDDO-Me) (EC_50_ = 0.05–0.08 ng/mL) (Figure 8A).

Compounds **1–3** activated Nrf2, with compound **1** being the most active with EC_50_ values of 116 nM (THP-1 cells), 202 nM (HEK001 cells), 268 nM (WS1 cells), and 385 nM (HMCB cells). However, although the compounds have agonistic activity on Nrf2, their EC_50_ values were higher than those of the CDDO-Me (EC_50_ = 0.08–0.13 nM) (Figure 8B).

### 2.6. Anti-Hyaluronidase Activity of the Extracts and Compounds of R. urticifolius

The inhibitory effects of *R. urticifolius* extracts on hyaluronidase were evaluated and they are shown in Table 2 in comparison to Quercetin, which exhibited an inhibition percentage of 95.72 ± 1.88% at an IC_50_ of 250.03 μg/mL. Among the examined extracts, DCMERu showed the highest hyaluronidase inhibition, reaching 72.54 ± 1.68% at an IC_50_ of 71.72 µg/mL. Conversely, AqERu (IC_50_ = 111.20 µg/mL) and HERu (IC_50_ = 165.30 µg/mL) had similar inhibition potential to DCMERu and the Quercetin, which showed inhibition percentages of 57.43 ± 1.92% and 36.71 ± 1.75%, respectively.

In the case of the hyaluronidase-inhibitory effects of compounds **1–3**, they exhibited inhibition percentages of 61.12 ± 1.78%, 30.45 ± 1.92%, and 16.12 ± 1.34% at IC_50s_ of 79.71 μM, 142.59 μM, and 171.97 μM, respectively. Thus, all compounds had higher activity than Quercetin, which had an inhibition percentage of 99.99 ± 1.19% at an IC_50_ of 820 μM.

### 2.7. Anti-Elastase and Anti-Collagenase Activities of the Extracts and Compounds of R. urticifolius

The inhibitory effects of *R. urticifolius* extracts on elastase and collagenase were evaluated, as shown in Table 3, and compared with Epigallocatechin gallate (EGCG), which had inhibition percentages of 94.44% ± 1.30 at an IC_50_ of 114.38 ± 3.07 μg/mL for elastase and 95.29% ± 2.09 at an IC_50_ of 114.04 ± 3.07 μg/mL for collagenase. Among the examined extracts, DCMERu showed the highest inhibition of elastase and collagenase, with percentages of 74.88% ± 1.5 at an IC_50_ of 30.42 ± 1.49 μg/mL for elastase and 90.31% ± 1.77 at an IC_50_ of 11.60 ± 1.69 μg/mL for collagenase.

In contrast, AqERu (57.09 ± 1.92 μg/mL) and HERu (102.35 ± 1.85 μg/mL) showed similar inhibition rates to DCMERu and EGCG. Their inhibition percentages for elastase were 52.86% ± 1.94 and 15.49% ± 1.76, respectively. For collagenase, AqERu showed an inhibition percentage of 52.86% ± 1.22 at an IC_50_ of 56.42 ± 1.32 μg/mL while HERu had an inhibition percentage of 36.53% ± 1.68 at an IC_50_ of 75.96 ± 1.96 μg/mL.

In the case of the elastase-inhibitory effects of compounds **1–3**, they had inhibition percentages of 60.76% ± 1.44, 17.68% ± 1.46, and 6.19% ± 1.44 at IC_50s_ of 80.13 ± 1.92, 168.11 ± 3.09, and 191.58 ± 3.78 μM, respectively. Regarding the collagenase-inhibitory effects of compounds **1–3**, they showed inhibition percentages of 62.24% ± 1.42, 24.87% ± 1.34, and 7.73% ± 1.21 at IC_50s_ of 76.59.02 ± 1.31, 152.40 ± 3.73, and 187.17 ± 2.61 μM, respectively.

All compounds exhibited higher activity than the EGCG, which had an inhibition percentage of 98.08% ± 1.74 at an IC_50_ of 250.37 ± 3.25 μM for elastase and 98.81% ± 1.42 at an IC_50_ of 250.54 ± 3.65 μM for collagenase.

## 3. Discussion

Analysing the extracts obtained through the different techniques used, we can indicate that the UV spectra obtained from the various extracts of *R. urticifolius* displayed a spectral pattern similar to that studied by Santacruz, who identified acylated and non-acylated anthocyanins in methanol–acetic acid extracts of the species *R. melagococcus* [36].

In the case of the ^1^H NMR spectra of the *R. urticifolius* extracts, although none showed considerable intensity between *δ*_H_ values of 8.5 and 11 ppm, the presence of aldehydes, ketones, and carboxylic acids cannot be ruled out. The presence of phenolic acids, flavonoids (quercetin, kaempferol, and apigenin), anthocyanins, terpenes (*E*-caryophyllene and rubusacid A), and alkaloids (indole and quinoline) with excellent antioxidant properties could be inferred based on comparisons with secondary metabolites from other Rubus species [37].

Following the analysis of the spectra and the corresponding identification of the compounds, we can state that these compounds have been previously described. For instance, considering the case of compound **1**, it is a derivative of phenacrylanilide and has been studied as a potential antifibrotic agent. However, as of 2024, no anti-aging effects of compound **1** have been reported [38]. Structurally, it is derived from the Tranilast drug, which contains a carboxylic acid, while compound **1** contains an *N*-methyl amide. Tranilast, known for its anti-allergic properties, has also shown potential therapeutic effects for psoriasis due to both its anti-inflammatory and anti-angiogenic properties as well as its ability to suppress collagen synthesis [39,40].

In the case of compound **2**, known as kynurenic acid (KYNA), this is a metabolite from the tryptophan pathway used in nutritional studies for individuals with vitamin B deficiencies [41]. KYNA has demonstrated beneficial effects as a neuroprotector, anticonvulsant, and analgesic [42,43,44]. Moreover, the pathway of this metabolite has been suggested to be crucial in determining the prognosis of COVID-19 [45]. KYNA’s association with the immune system, inflammation, and cancer has become increasingly apparent [46]. Notably, this was the first time kynurenic acid was isolated from a plant species as it was previously considered part of animal metabolism and *Saccharomyces cerevisiae* [47].

Finally, compound **3**, known as α-Cyano-4-hydroxycinnamic acid (α-CCA), has been recognised for its anti-tyrosinase activity and its impact on aldose reductase, suggesting potential benefits for Alzheimer’s disease [48,49]. Furthermore, it has shown effectiveness in murine in vivo and in vitro breast cancer models [50].

Once the compositions of the extracts were analysed, their cytotoxic effects were determined in vitro on different cell lines. The three extracts of *R. urticifolius* studied in this work did not exhibit cytotoxicity. These findings were consistent with the existing literature. Although the genus Rubus includes more than 700 plant species, there have been no previous reports of toxicity in the *R. urticifolius* species. Numerous studies on cell viability have shown that hydroalcoholic extracts from different Rubus species do not exhibit toxicity, making them suitable for human use. For example, the hydroalcoholic extract of *R. coreanus* has not shown significant toxicity below 300 µg/mL in LPS assays [51]. Similarly, the hydroalcoholic extract of *R. niveus* has not shown cytotoxicity below 200 mg/kg [52]. In terms of the viability results of the *R. urticifolius* extracts, none of the three extracts showed higher cytotoxicity than ACTD. This can be attributed to their chemical composition, which is rich in highly lipophilic compounds that can penetrate cell membranes more effectively [53].

As for the isolated compounds, although previous studies have reported no cytotoxicity for compound **1** (clogP = 2.07), its cytotoxicity can be attributed to the lipophilicity conferred by its fragments of aromatic ring and methylated alcohols [54]. As for compound **2**, previous cytotoxicity studies in HaCaT cells did not show statistically significant cytotoxic effects at a concentration of 21.51 mM [55]. Additionally, the low toxicity of compound **2** was also previously demonstrated by Turski et al. [56], who showed that diets containing large amounts of compound **2** (25–250 mg/L) or enriched with it for 3–21 days posed no risk to human health. In our case, the decrease in the cytotoxicity of compound **2** (clogP = 1.71) could have been due to the presence of a non-methylated alcohol group and an acidic group in its structure, which would increase its hydrophilic capacity (slightly less polar), decreasing its cellular permeability [57]. Lastly, there are no reports on the cytotoxicity of compound **3**. However, the significant decrease in the cytotoxicity of compound **3** (clogP = 1.44) can be attributed to the presence of an alcohol group, an acidic group similar to compound **2**, and a cyanide group, which further increases its hydrophilic character (being the least polar of the three compounds) and hinders its passage through cell membranes [57].

Following the chemical analysis and cytotoxicity determination of the samples, we proceeded to discuss the conducted biological activities. The anti-melanogenic activity of other species of the Rubus genus has been reported, such as for *R. fraxinifolius*, whose methanolic extract has exhibited anti-melanogenic activity in addition to showing antioxidant, anti-elastase, and anti-skin-aging activities [58]. When analysing the melanin- and tyrosine-inhibitory capacities of *R. urticifolius* extracts, we observed that DCMERu had a higher inhibitory effect, followed by AqERu and HERu, which could have been due to the composition of compounds extracted with dichloromethane/methanol that are similar to those extracted with methanol from the *R. fraxinifolius* species.

In this regard, compound **1** can interact with specific residues in the active site of tyrosinase (amino acids such as histidine and cysteine) through functional groups present in its structure, affecting the enzyme’s geometry and reducing its catalytic activity [59]. These interactions can alter the enzymatic activity of tyrosinase and block the conversion of tyrosine to dopaquinone, a key step in melanin synthesis [60]. Additionally, compound **1** can exert competitive inhibition of tyrosinase, competing with the substrate tyrosine for binding to the enzyme’s active site. Besides its direct effects on tyrosinase, it is suggested that compound **1** can modulate intracellular signalling pathways such as the MAPK and NF-κB pathways, known for their role in regulating the expression of genes related to melanogenesis [61].

To date, no studies have reported the inhibitory activity of compound **2** on melanin production and tyrosinase activity, but by sharing a structural similarity with Kynurenine, a precursor in the Kynurenine metabolic pathway, it can have effects on reducing melanin content levels in melanocytes and keratinocytes [62]. In this regard, compound **2** can interfere with melanin biosynthesis by competing with the Kynurenine pathway for tryptophan, an essential amino acid in both metabolic pathways. This process can involve competition for shared enzymes or the formation of complexes with specific enzymes involved in the Kynurenine pathway, reducing the availability of tryptophan for melanogenesis [63].

Finally, in the case of compound **3,** there have been no reports of its inhibitory activity on melanin production and tyrosinase activity. However, the anti-tyrosinase activity of structurally related compounds has been quantified [64]. Among these compounds, we highlight 4-hydroxycinnamic acid, whose only structural difference from compound **3** is the absence of a cyanide group. In this sense, compound **3** can exert the competitive inhibition on tyrosinase, competing with the substrate tyrosine for the enzyme’s active site, blocking the conversion of tyrosine to dopaquinone (a critical step in melanin synthesis). The presence of the cyano functional group is relevant as it forms hydrogen bonds or dipole interactions with specific residues in the active site of tyrosinase (histidine and cysteine residues) [65].

Although there have been no previous reports on *R. urticifolius*, other species of the Rubus genus have shown anti-inflammatory properties. For example, the extract of *R. coreanus* inhibits NF-κB by preventing the phosphorylation of IκB, with an IC_50_ of 200 µg/mL [51]. The degree of anti-inflammatory activity in *R. coreanus* depends on the fruit’s ripeness [66]. Additionally, extracts of *R. idaeus* have demonstrated in vivo anti-inflammatory efficacy by reducing oedema at a dose of 130 mg/kg [67].

The anti-inflammatory activity in the Rubus genus is related to the presence of anthocyanins and gallic acid. Extracts rich in anthocyanins have been found to inhibit pro-inflammatory factors such as NF-kB, TNF-α, NO, MAPK, IKK, IκBα, and COX-2 in macrophages [68,69].

In the case of the anti-inflammatory activity of the compounds, no studies have been reported on the anti-inflammatory activity of compound **1**. However, its anti-inflammatory activity could be related to caffeic acid phenethyl ester (CAPE) because it shares part of its structure [70]. Therefore, compound **1** could interfere with the activity of IKK (IκB kinase), which is responsible for phosphorylating IκBα. By preventing the phosphorylation of IκBα, compound **1** will stabilise the IκBα-NF-κB complex in the cytoplasm, preventing NF-κB from translocating to the nucleus [71]. Additionally, the presence of the acryloyl amide group in compound **1** allows it to form hydrogen bonds or π–π stacking interactions with specific residues in the p65 subunit of NF-κB, stabilising a complex that prevents p65 from binding to DNA, thereby blocking the activation of NF-κB-dependent gene transcription [72].

Regarding compound **2**, although there are no reports on its anti-inflammatory activity, its anti-inflammatory potential can be compared to that of very similar compounds such as KYNA [73]. In this regard, the specific interactions between compound **2** and the proteins involved in the NF-κB pathway can form hydrogen bonds through the carboxylic acid group and the hydroxyl group. These functional groups can interact with IKK (serine residues) and the p65 subunit (lysine and arginine residues) of NF-κB, stabilising conformations that prevent NF-κB activation [74]. Additionally, due to the quinoline-like structure of compound **2**, this compound can lead to van der Waals interactions with the hydrophobic surfaces of proteins, contributing to the inhibition of NF-κB [75].

Finally, the anti-inflammatory activity of compound **3** has been studied by comparing it with the 4-hydroxycinnamic acid, with which it shares a similar structure, except for the presence of an additional cyano group. This cyano group would allow compound **3** to form hydrogen and electrostatic bonds with critical residues in IKK such as lysine and serine, blocking its NF-κB activity. This would prevent the phosphorylation of IκBα, avoiding its degradation and keeping NF-κB inactive in the cytoplasm [76]. Additionally, the presence of the cyano group in compound **3** can form additional hydrogen bonds and π–π stacking interactions with aromatic residues in p65, stabilising an inactive conformation of p65, thereby preventing NF-κB binding to DNA and consequently blocking the transcription of NF-κB-dependent genes [77].

Several studies have described the phytochemical compositions of hydroalcoholic extracts from fruits of the Rubus genus. All of them agree that Rubus extracts are rich in polyphenolic compounds such as glycosylated cyanidins, catechins, saponins, and phenolic acids. These compounds are responsible for the antioxidant potential of the extracts [78,79].

In the case of *R. urticifolius*, the antioxidant capacity of the alcoholic extract has been reported at a concentration of 15.60 mg GAE/g of fresh fruit [80] or an EC_50_ of 22.78 µg/mL in the DPPH assay, with an antioxidant activity of 81% [34]. These results support our findings of Nrf2 activation by those extracts containing polyphenolic compounds. Furthermore, the quantity of cyanidins and other phenolic compounds is higher in extracts from immature fruits than in mature ones. Therefore, consuming green fruits is more beneficial as their therapeutic effect is more potent [34].

About the isolated compounds, compound **1** was the most potent. This is attributed to the presence of an acryloyl amide fragment in its chemical structure, which suggests the presence of electrophilic sites capable of interacting through adducts with specific cysteine thiol groups in Keap1. These interactions induce conformational changes that inhibit Keap1’s ability to target Nrf2 for degradation [81]. By modifying Keap1, compound **1** prevents the degradation of Nrf2, allowing its accumulation and translocation to the nucleus, where it activates the transcription of ARE-driven genes, thus enhancing the skin’s defence against oxidative stress [82].

This mechanism of action also applies to compound **3**, which contains an acrylic acid in its structure. However, the difference in activity between these compounds is due to other factors such as lipophilicity. Compound **1** has a LogP of 3.24, making it more lipophilic and facilitating its accumulation in the cell, whereas compound **3** has a much lower LogP of 0.85, reducing its cellular permeability. In the case of compound **2**, with a LogP of 1.07, the mechanism of action on Nrf2 is different. Due to its quinoline-like structure and the presence of a hydroxyl group, compound **2** can directly capture ROS, thereby reducing oxidative stress. This reduction in oxidative stress indirectly promotes the activation of Nrf2, facilitating its translocation from the cytoplasm to the nucleus and promoting the expression of antioxidant genes such as SOD1 and GPx [83,84].

It is always important to note that the activation of Nrf2 signalling is not always beneficial as it can promote cancer progression and chemoresistance [85]. This consideration is crucial, especially in patients with cancer who are undergoing chemotherapy, particularly with platinum-based chemotherapeutics. The inappropriate activation of Nrf2 in these contexts can be very dangerous as it could protect cancer cells from the oxidative stress induced by chemotherapeutic treatments, thereby reducing their efficacy. Therefore, any therapeutic application of compounds that activate Nrf2 signalling must be carefully evaluated to avoid adverse effects in cancer patients.

Previous studies conducted by Hering et al. [86] demonstrated that the aqueous and ethanolic extracts of *R. caesius* possess anti-hyaluronidase activity, with IC_50_ values of 55.24 ± 3.21 and 68.7 ± 1.61 μg/mL, respectively. Likewise, Marquina et al. [87] determined that the aqueous extract of *R. fruticosus* has a 40% inhibition capacity of hyaluronidase. Additionally, Küpeli et al. [88] demonstrated that the DMSO extract of *R. sanctus* inhibited hyaluronidase by 20% at a concentration of 100 µg/mL, as measured by the amount of *N*-acetylglucosamine released by sodium hyaluronate. Araujo et al. [89] reported the anti-hyaluronidase activity of *R. fruticosus* by measuring the amount of *N*-acetylglucosamine released by potassium hyaluronate due to the phenolic compounds present in this species. Finally, Nakahara et al. [90] reported that the aqueous, 60% ethanolic, and 70% acetonic extracts of *R. suavissimus* inhibited hyaluronidase with IC_50_ values of 140, 45, and 25 µg/mL, respectively.

In our case, the DMCERu showed higher anti-hyaluronidase activity than the AqERu and the HERu, which did not exhibit relevant inhibitory activity. This could be due to the composition of compounds extracted with dichloromethane/methanol, similar to those extracted with ethanol, acetone, and DMSO from *R. caesius, R. suavissimus*, and *R. sanctus* species, respectively.

To date, no studies have been conducted on the anti-hyaluronidase activity of compound **1**. However, its basic phenylpropanoid structure is similar to that of other molecules with anti-hyaluronidase activity, such as CAPE or ferulic acid. Osés et al. [91] reported that the propolis extract, containing CAPE and ferulic acid, inhibited hyaluronidase by up to 68% at a concentration of 10 mg/mL. Additionally, Gębalski et al. [92] found that methoxy groups at positions C3′ and C4′ in phenylpropanoid molecules increased anti-hyaluronidase activity. For example, derivatives of Rosmarinic acid showed hyaluronidase inhibition values of up to 50% at concentrations between 183 and 1049 μM [93]. Therefore, compound **1**, as a phenylpropanoid, could inhibit hyaluronidase by interacting with its active site and due to its antioxidant and anti-inflammatory properties.

Compound **1** also has an almost identical structure to Tranilast, a known hyaluronidase inhibitor. Kakegawa et al. [94] demonstrated that Tranilast inhibited 75% of active hyaluronidase at a concentration of 2 mM. However, when an activator was added, Tranilast inhibited 50% of active hyaluronidase at the same concentration. The structural difference between Tranilast and compound **1** (the change from a carboxyl group in Tranilast to a carboxamide group in compound **1**) could make compound **1** more potent in hyaluronidase inhibition at lower concentrations. Kaessler et al. [95] compared hyaluronidase inhibition by carboxamide and acetamide indoles, observing that molecules with carboxamide chains exerted higher inhibition at a concentration of 50 μM. The inhibition percentage of carboxamide molecules was 50% while that of acetamide molecules was 10%. Therefore, the carboxamide chain in compound 1 contributes to its higher anti-hyaluronidase activity.

No specific studies have been conducted on the anti-hyaluronidase activity of compound **2**, but its quinoline structure suggests inhibitory potential. Osorio et al. [96] determined the anti-hyaluronidase activity of quinoline–hydrazone hybrids, reporting a 60% inhibition at a concentration of 250 μM. This activity was corroborated by molecular docking studies observing that oxygen and carbon atoms in the quinoline ring formed hydrogen bonds or hydrophobic interactions with key residues in the active site of hyaluronidase, thus blocking its catalytic activity. Therefore, compound **2**, with its quinoline structure, could have a similar mechanism of action.

The anti-hyaluronidase activity of compound **3** is attributed to its cinnamic acid structure. Do Prado et al. [97] obtained bioactive compounds from fermented soy, finding that *trans*-cinnamic acid inhibited hyaluronidase by more than 60%. Thippeswamy and Rajeshwara [98] also found that cinnamic acid inhibited hyaluronidase with an IC_50_ of 336 µg/mL. Since compound **3** structurally resembles *trans*-cinnamic acid, it could inhibit hyaluronidase by interacting with its cyanide and phenolic hydroxyl groups at the enzyme’s active site through hydrogen bonds or hydrophobic interactions.

Previous studies have researched the inhibitory properties of elastase and collagenase in species of the genus Rubus. For example, methanolic leaf extracts of *R. fraxinifolius* exhibited anti-elastase activity with an IC_50_ value of 57.45 μg/mL [99]. Similarly, ethanolic extracts of ripe berries of *R. occidentalis* showed elastase inhibition values of 8.8% and 15.9% at concentrations of 100 μg/mL and 300 μg/mL, respectively, and collagenase inhibition of 16.4% at a concentration of 300 μg/mL [100]. Similarly, the extract of *R. nigrum* showed collagenase inhibition of 8.28% at a concentration of 300 μg/mL [101].

Analysing the inhibitory activity of *R. urticifolius* extracts on elastase and collagenase, it was found that the DCMERu had higher activity than the AqERu and the HERu. This could have been due to the possible composition of compounds extracted with dichloromethane/methanol that may be similar to those extracted with methanol or ethanol from *R. fraxinifolius* and *R. occidentalis* species, respectively.

In the case of isolated compounds, the anti-elastase and anti-collagenase actions of compound **1** have not been directly studied. However, its structural similarity to CAPE suggests that compound **1** could interact through hydrogen bonding and possibly hydrophobic interactions with the active site (serine, histidine, and aspartate) of elastase, blocking substrate access to the enzyme and thus preventing the breakdown of elastic fibres in connective tissues [102]. The inhibition of collagenase by compound **1** would involve chelation formation at the active site of collagenase, interfering with its involvement in the catalytic reaction. Additionally, compound **1** could interact with specific catalytic residues (histidine and glutamate) at the active site of collagenase, blocking the activation of water necessary for collagen hydrolysis [103].

Furthermore, no studies have explored the inhibitory activity of compound **2** on elastase or collagenase. However, since compound **2** is a derivative of quinoline, its ability to inhibit these enzymes can be suggested based on its chemical nucleus [104]. The quinoline ring of compound **2** could interact with the active sites of elastase and collagenase due to its aromatic structure and the presence of nitrogen atoms in the ring. Additionally, the hydroxyl group at position 4 of compound **2** could form interactions (hydrogen bonds) with specific residues in the active site of these enzymes, resulting in the inhibition of their activity. Finally, the presence of the carboxyl group at position 2 of the quinoline could allow compound **2** to interact with metal ions in the active site of collagenase through chelation, interfering with the enzyme’s catalytic activity [98].

Compound **3**, a member of the hydroxycinnamic acid family, shares structural similarities with compounds that show collagenase inhibition, such as 3,4,5-trihydroxycinnamic acid (THCA) [105]. The cyano and phenolic hydroxyl groups of compound **3** could participate in the formation of hydrogen bonds with key residues in the active site, contributing to the inhibition of elastase and collagenase activity [106]. Additionally, like 3,4,5-trihydroxycinnamic acid, compound **3** could chelate the metal ions necessary for the catalytic activity of collagenase, interfering with the enzyme’s ability to hydrolyse collagen [105].

In summary, the mechanism of action of these compounds may involve inhibiting tyrosinase activity, thereby reducing melanin synthesis. A reduction in melanin production would influence NF-κB signalling as melanogenesis intermediates can modulate NF-κB activity. Additionally, reducing melanin production can decrease oxidative stress in the skin, leading to the decreased degradation of Nrf2 by Keap1 and increased translocation of Nrf2 to the nucleus, activating the transcription of antioxidant and anti-inflammatory genes. Finally, the modulation of NF-κB and the activation of Nrf2 by the compounds may have indirect effects on the activity of hyaluronidase, elastase, and collagenase as reducing oxidative stress and inflammation in the skin can influence the activity of these enzymes.

## 4. Materials and Methods

### 4.1. General Experimental Procedures

High-quality organic solvents from Merck were used to extract the compounds from *R. urticifolius*. Thin-layer chromatography (TLC) was performed using silica gel (Silica gel 60 GF254 Merck, Cat. No. 112926-00-8, St. Louis, MO, USA). The samples were analysed using both phosphomolybdic acid solution (12Mo_12_O_3_·H_3_PO_4_ ≥ 99.99% Merck, Cat. No. 51429-74-4, St. Louis, MO, USA) and visualisation through a UV light (Spectroline^®^ E-Series UV lamp with a 254 nm wavelength, 230 V, New York, NY, USA). For column chromatography, silica gel with a particle size of 40–63 µm (SiO_2_ Merck, Cat. No. 112926-00-8, St. Louis, MO, USA) was used with the eluents specified in the extraction and isolation section. Nuclear magnetic resonance (NMR) experiments were conducted with a Bruker Avance DRX 300 spectrometer, operating at 300 MHz (^1^H) and 75 MHz (^13^C), using deuterated methanol (CD_3_OD, 99.8 atom % D, Merck, Cat. No 811-98-3, DA, DE) and deuterated dimethyl sulfoxide ((CD_3_)_2_SO, 99.8 atom % D, Merck, Cat. No 2206-27-1, DA, DE) as the deuterated solvents. High-resolution electron ionisation mass spectrometry (HREIMS) was carried out with a Bruker MAXIS II spectrometer, utilising the electrospray ionisation technique (EI^+^). Samples were directly infused at a flow rate of 3 μL/min with methanol (MeOH 99.8%, Merck, Cat. No. 67-56-1, St. Louis, MO, USA) containing 0.1% formic acid (HCOOH 97.5–98.5%, Merck, Cat. No. 64-18-6, St. Louis, MO, USA) as the ionising phase. Initial parameters included an end plate at 500 V, capillary at 3500 V, nebuliser at 0.2 bar, dry gas at 2.0 L/min, dry temperature at 250 °C, and a mass range of 50–3000 Da.

### 4.2. Extraction and Isolation

Fruits of *R. urticifolius* were collected in June 2019 from the San Juan Huancollo Community in the Murillo Province, La Paz Department, Bolivia 16°3′15.1″ S 68°5′21.2″ W), at an altitude of 3918 m. Botanical identification was confirmed by Dr. Carla Maldonado at the National Herbarium of Bolivia and a voucher specimen (48393) was deposited.

Upon collection, the plant roots were dried in a hot air oven at 50 °C for 48 h and pulverised into a fine powder (500 g). This powder underwent a 30 min decoction at boiling point with 2 L of distilled water (DH_2_O). The resulting aqueous extract (AqERu) was frozen in glass containers at −38 °C and subsequently lyophilised using a freeze dryer (Christ alpha 1e2 LD plus, Osterode am Harz, Germany) at −50 °C.

After being lyophilised, the sample (61.51 g) underwent three extractions with 500 mL of *n*-hexane (Hex, Merck, Cat. No. 110-54-3, St. Louis, MO, USA) at room temperature (25 ± 5 °C) for 72 h and then evaporated in vacuo, yielding 8.18 g of the *n*-hexane extract of *R. urticifolius* (HERu). Subsequently, three additional extractions with 500 mL (1:1 v/v) of dichloromethane (DCM, ≥ 99.5% Merck, Cat. No. 75-09-2, St. Louis, MO, USA)/MeOH were carried out under similar conditions, yielding 4.93 g of the dichloromethane/methanol extract of *R. urticifolius* (DCMERu).

DCMERu (4 g) was fractionated using bio-guided silica gel (40–63 μm) column chromatography with a Hex/Ethyl acetate (AcOEt 99.8% Merck, Cat. No. 141-78-6, St. Louis, MO, USA) gradient (9:1 → 0:10). Fourteen fractions (F1 → F14) were obtained, with F4 (172.67 mg), F7 (159.34 mg), and F11 (151.02 mg) showing the highest activities across all tested cell lines.

Fraction F4 (150 mg) was chromatographed using a silica gel column (40–63 μm) (Hex/AcOEt 8:2 → 2:8), yielding eight subfractions (F4A → F4H). Subfraction F4D (Compound **1**, 21.02 mg) showed promising activities.

The process was repeated with fraction F7 (150 mg), separated by silica gel column chromatography (40–63 μm) (MeOH/AcOEt (2:8 → 5:5)), yielding six subfractions (F7A → F7F). Subfraction F7B (Compound **2**, 30.24 mg) exhibited the most activity.

Finally, chromatography was repeated with fraction F11 (150 mg), separated by silica gel column chromatography (40–63 μm) (MeOH/DCM, 0.5:8.5 → 0:10), yielding nine subfractions (F11A → F11I). Subfraction F11G (Compound **3**, 25.01 mg) showed the best results.

### 4.3. Spectroscopic Data

#### 4.3.1. (E)-2-(3-(3,4-Dimethoxyphenyl)acrylamido)-N-methylbenzamide (**1**)

Characteristics: white solid, ^1^H NMR (500 MHz, DMSO-*d_6_*) *δ_H_*: 8.75 (s, 1H, H-6′), 8.57 (d, *J* = 8.3 Hz, 1H, H-5′), 7.74 (dd, *J* = 7.9, 1.4 Hz, 1H, H-2′), 7.54 (d, *J* = 15.4 Hz, 1H, H-2), 7.50 (d, *J* = 8.6 Hz, 1H, H-6), 7.39 (s, 1H, NH), 7.24 (dd, *J* = 8.3, 1.9 Hz, 1H, H-8), 7.15 (t, *J* = 7.6 Hz, 1H, H-9), 6.99 (d, *J* = 8.3 Hz, 1H, H-7), 6.76 (d, *J* = 15.5 Hz, 1H, H-3), 3.85 (s, 3H, -OMe), 3.80 (s, 3H, -OMe), 2.83 (d, *J* = 3.8 Hz, 3H, -NMe); ^13^C NMR (126 MHz, DMSO-*d_6_*) *δ_C_*: 169.19 (CONH), 164.40 (C-1), 151.05 (C-6), 149.46 (C-7), 141.86 (C-3), 139.70 (C-1′), 132.23 (C-5′), 128.39 (C-3′), 127.77 (C-4), 123.19 (C-6′), 123.03 (C-9), 121.20 (C-4′), 121.16 (C-2′), 120.40 (C-2), 111.97 (C-8), 110.68 (C-5), 56.11 (-OMe), 55.99 (-OMe), 26.79 (-NMe); HREIMS *m*/*z* [M + H]^+^ 341.1479. Data were compared to the references [38].

#### 4.3.2. 4-Hydroxyquinoline-2-carboxylic Acid (**2**)

Characteristics: white powder; ^1^H NMR (500 MHz, Methanol-*d_4_*) *δ_H_:* 8.26 (dd, *J* = 8.2, 1.4 Hz, 1H, H-5), 7.87 (d, *J* = 8.6 Hz, 1H, H-8), 7.76 (ddd, *J* = 8.5, 6.9, 1.5 Hz, 1H, H-6), 7.48 (ddd, *J* = 8.1, 6.9, 1.1 Hz, 1H, H-7), 7.00 (s, 1H, H-3); ^13^C NMR (126 MHz, Methanol-*d_4_*) *δ_C_:* 176.01 (C-4), 163.90 (COOH), 142.32 (C-9), 140.35 (C-2), 131.65 (C-7), 125.22 (C-10), 124.73 (C-8), 123.83 (C-5), 121.31 (C-6), 109.05 (C-3); HREIMS *m*/*z* [M + H]^+^ 190.0495 (calculated for C_10_H_8_NO_3_, 190.1780). Data were compared to the references [107].

#### 4.3.3. (E)-2-Cyano-3-(4-hydroxyphenyl)acrylic Acid (**3**)

Characteristics: white powder; ^1^H NMR (500 MHz, Methanol-*d_4_*) *δ_H_:* 8.17 (s, 1H, H-3), 7.94 (s, 1H, H-2′), 7.92 (s, 1H, H-6′), 6.91 (s, 1H, H-3′), 6.90 (s, 1H, H-5′); ^13^C NMR (126 MHz, Methanol-*d_4_*) *δ_C_:* 165.90 (C-1), 164.02 (C-4′), 155.82 (C-3), 134.87 (C-2′/C-6′), 124.45 (C-1′), 117.62 (-CN), 117.16 (C-3′/C-5′), 99.46 (C-2); HREIMS *m*/*z* [M + H]^+^ 190.0499 (calculated for C_10_H_8_NO_3_, 190.0504). Data were compared to the references [108].

### 4.4. Cell Culture

The HEK001 (Homo sapiens cutaneous keratinocyte, CCL-2404), WS1 (*Homo sapiens* cutaneous fibroblast, CRL-1502), HMCB (*Homo sapiens* cutaneous melanoma, CRL-9607), and THP-1 (*Homo sapiens* peripheral blood monocyte, TIB-202) cell lines used in this study were obtained from the American Type Culture Collection (ATCC, Manassas, VA, USA). Cultivated in a Roswell Park Memorial Institute (RPMI) 1640 medium (RPMI 1640 Merck, Cat. No. R7755, New York, NY, USA) supplemented with 2 mM L-glutamine (≥99% Merck, Cat. No. 56-85-9), 10% fetal bovine serum (FBS Merck, Cat. No. TMS-016, St. Louis, MO, USA), and 10,000 units of penicillin and 10 mg streptomycin/mL (Merck, Cat. No. P4333, St. Louis, MO, USA) in culture flasks, the cells were maintained in an incubator under normoxic conditions (20–21% O_2_) and a humidified atmosphere (5% CO_2_ at 37 °C).

Mother solutions of samples (extracts, fractions, and compounds) were prepared at a concentration of 1 mM using dimethyl sulfoxide (DMSO ≥ 99.9% Merck, Cat. No. 67-68-5, St. Louis, MO, USA) as the solvent. From these mother solutions, dilutions were prepared at concentrations ranging from 100 to 0.20 μg/mL or μM in culture medium containing 0.5% DMSO. Additionally, a negative control group consisting solely of culture medium with 0.5% DMSO was included to assess its potential cytotoxic effect on the cells.

### 4.5. Statistical Analysis

Data were analysed using Prism v9.0.0 (GraphPad Software LLC, San Diego, CA, USA, 1994–2020). After normalisation, the data were plotted, and the percentage of activity was calculated in relation to the logarithm of the compound concentration. The 50% cytotoxic concentration (CC_50_), 50% inhibitory concentration (IC_50_), and half-maximal effective concentration (EC_50_) values were determined using a sigmoidal dose–response curve. Additionally, a one-way ANOVA with ‘Tukey’s multiple comparisons test was performed to evaluate the statistical significance of differences between the values (*p* < 0.05; *p* < 0.001).

### 4.6. In Vitro Viability Assay

Cell viability was evaluated using a colorimetric assay in 96-well plates, employing 3-amino-7-dimethylamino-2-methylphenazine hydrochloride salt reagent (NR, Merck, Cat. No. 553-24-2, St. Louis, MO, USA) [109]. Actinomycin D (ACTD ≥ 95% Merck, Cat. No. 50-76-0, St. Louis, MO, USA) was used as a positive control with an IC_50_ of 0.01 μg/mL for the extracts and fractions, equivalent to 0.008 μM for the compounds.

HEK001, WS1, HMCB, and THP-1 cell lines were cultured in 96-well plates at 37 °C with 5% CO_2_ until > 90% confluency was achieved (1 × 10^6^ cells/well). Subsequently, the cultures were treated with sample dilutions (100–0.20 μg/mL or μM) for 72 h at 37 °C with 5% CO_2_ in phenol red-free RPMI. The supernatant was then discarded and the cells were washed with phosphate-buffered saline (PBS) before being incubated with 100 μM NR (50 μg/mL; 173 μM) for 3 h at 37 °C with 5% CO_2_. NR labelling was performed after DMSO treatment. After incubation, the labelled sample dilutions were rapidly washed with a fixative solution composed of 1% calcium chloride (CaCl_2_ Merck, Cat. No. 10043-52-4, St. Louis, MO, USA) and 0.5% formaldehyde solution (≥36.0% in H_2_O Merck, Cat. No. 50-00-0, St. Louis, MO, USA), then diluted with 100 μL of the NR extraction solution. Finally, the plates were read at 540 nm using a microplate reader (Anthos 2020, version 2.0.5, Biochrom Ltd., Cambridge, UK).

### 4.7. Cellular Tyrosinase Assay

To determine the anti-tyrosinase activity of the samples (at the same concentration as in the viability assay), tyrosinase inhibition assay was used, following the protocol carried out by Apaza Ticona et al. [110]. HMCB cells were cultured in 96-well plates (1 × 10^6^ cells/well). Kojic acid (Merk, Cat. No. 501-30-4, St. Louis, MO, USA) was used as a positive control with an IC_50_ of 1.97 μg/mL for the extracts and fractions, equivalent to 13.9 μM for the compounds. Absorbance was measured at 475 nm using a microplate reader.

### 4.8. Melanin Content Assay

To determine the anti-melanogenic activity of the samples (at the same concentration as in the viability assay), melanin inhibition assay was used, following the protocol carried out by Apaza Ticona et al. [110]. HMCB cells were cultured in 96-well plates (1 × 10^6^ cells/well). Kojic acid (Merk, Cat. No. 501-30-4, St. Louis, MO, USA) was used as a positive control with an IC_50_ of 1.97 μg/mL for the extracts and fractions, equivalent to 13.9 μM for the compounds. Absorbance was measured at 405 nm using a microplate reader.

### 4.9. NF-κB Inhibition Assay

To determine the anti-inflammatory activity of the samples (at the same concentration as in the viability assay), NF-κB inhibition assay was used, following the protocol carried out by Apaza Ticona et al. [111]. Cells were cultured in 96-well plates (1 × 10^6^ cells/well). Celastrol (≥98% Merk, Cat. No. 34157-83-0, St. Louis, MO, USA) was used as a positive control with an IC_50_ of 3.34 μg/mL for the extracts and fractions, equivalent to 7.41 μM for the compounds. Absorbance was measured at 450 nm using a microplate reader.

### 4.10. Nrf2 Activity Assay

To determine the antioxidant response of the samples (at the same concentration as in the viability assay), Nrf2 activation assay was used, following the protocol carried out by Apaza Ticona et al. [111]. Cells were cultured in 96-well plates (1 × 10^6^ cells/well). 2-Cyano-3,12-dioxo-oleana-1,9(11)-dien-28-oic acid methyl ester (CDDO-Me ≥98% Merk, Cat. No. 218600-53-4, St. Louis, MO, USA) was used as positive control with an EC_50_ of 0.07 ng/mL for the extracts and fractions, equivalent to 0.11 nM for the compounds. Absorbance was measured at 540 nm using a microplate reader.

### 4.11. Hyaluronidase Inhibition Assay

The hyaluronidase-inhibitory effect was evaluated, implementing the protocol adapted from Sahasrabudhe and Deodhar [112]. Quercetin (≥95% Merck, Cat. No. 117-39-5, St. Louis, MO, USA) was used as positive control with an IC_50_ of 250 μg/mL for the extracts and fractions, equivalent to 820 μM for the compounds.

A 0.1 M acetate buffer at pH 3.6 was prepared and used as the buffer solution for this assay. Initially, 10 μL of 8 mg/mL hyaluronidase in buffer solution and the samples at the same concentrations used as in the viability assay were mixed in a test tube and incubated at 37 °C for 20 min. Then, 20 μL of 12.5 mM calcium chloride (CaCl_2_ ≥ 96.0% Merck, Cat. No. 10043-52-4, St. Louis, MO, USA) was added to the mixture and incubated at 37 °C for another 20 min. After incubation, the activated Ca^2+^ mixture was treated with 50 μL of 2.4 μg/mL hyaluronic acid (HA Merck, Cat. No. 9004-61-9, St. Louis, MO, USA) in buffer and further incubated at 37 °C for 40 min. The reaction mixture developed colour upon adding 2 μL of 0.4 N sodium hydroxide (NaOH pellets ≥ 97.0%, Merck, Cat. No. 1310-73-2, St. Louis, MO, USA) and 20 μL of 0.4 N potassium tetraborate tetrahydrate (K_2_B_4_O_7_·4H_2_O ≥ 99.5% Merck, Cat. No. 12045-78-2, St. Louis, MO, USA), followed by incubation in a water bath at 100 °C for 3 min. A solution of 4-(dimethylamino)benzaldehyde (DMAB 99% Merck, Cat. No. 100-10-7, St. Louis, MO, USA) was prepared, containing 0.4 g of DMAB dissolved in 35 mL of 100% acetic acid (AcOH ≥ 99.7% Merck, Cat. No. 64-19-7, St. Louis, MO, USA) and 5 mL of 10 M hydrochloric acid (HCl 37% Merck, Cat. No. 7647-01-0, St. Louis, MO, USA). Finally, 600 μL of DMAB solution was added to the mixture after cooling to room temperature (25 ± 5 °C) and incubated at 37 °C for 20 min. Absorbance was measured at 585 nm using a microplate reader.

### 4.12. Elastase-Inhibitory Effect Assay

The elastase-inhibitory effect was evaluated, implementing the protocol adapted from Tu and Tawata [113]. Epigallocatechin gallate (EGCG, ≥ 95%, Merck, Cat. No. 989-51-5, St. Louis, MO, USA) was used as positive control with an IC_50_ of 114 μg/mL for the extracts and fractions, equivalent to 250 μM for the compounds.

A mixture containing *N*-succinyl-Ala-Ala-Ala-*p*-nitroanilide (AAAPVN, Merck, Cat. No. 52299-14-6, St. Louis, MO, USA) elastase substrate in 0.1232 M Tris-HCl buffer solution (pH 8) was prepared to achieve a concentration of 1.015 mM. This elastase substrate was combined with 10 μL of the test samples at the same concentrations used as in the viability assay in 96-well plates and pre-incubated at room temperature (25 ± 5 °C) for 10 min. Subsequently, the reaction was initiated by adding 10 μL of elastase from porcine pancreas (7.5 units/mL) in Tris buffer solution to the pre-incubated mixtures. Absorbance was then measured at 410 nm using a microplate reader.

### 4.13. Collagenase Inhibitory Assay

The hyaluronidase-inhibitory effect was evaluated, implementing the protocol adapted from Wang et al. [114]. Epigallocatechin gallate (EGCG, ≥ 95%, Merck, Cat. No. 989-51-5, St. Louis, MO, USA) was used as positive control with an IC_50_ of 114 μg/mL for the extracts and fractions, equivalent to 250 μM for the compounds.

A fixed weight of 1 mg of azo dye-impregnated collagen was measured in the test tubes. Subsequently, 800 μL of 0.1 M Tris-HCl (pH 7) and 100 μL of the samples at the same concentrations used as in the viability assay were added to each test tube. Next, 100 μL of collagenase (200 units/mL) was immediately mixed into the mixture and the tubes were incubated at 43 °C for 1 h. After incubation, the test tubes were centrifuged at 3000 rpm for 10 min. The supernatant from each tube was transferred into 96-well plates and the absorbance of each supernatant was measured at 550 nm using a microplate reader.

## 5. Conclusions

This study represented a significant advancement, being the first report to document the isolation and characterisation of (*E*)-2-(3-(3,4-dimethoxyphenyl)acrylamido)-*N*-methylbenzamide (**1**), 4-hydroxyquinoline-2-carboxylic acid (**2**), and (*E*)-2-cyano-3-(4-hydroxyphenyl)acrylic acid (**3**) from the *Rubus urticifolius* species. These findings underscore the importance of investigating the therapeutic potential of this plant and its bioactive compounds.

The results demonstrate the promising therapeutic potential of the aqueous extract of *Rubus urticifolius*, particularly in terms of safety, with cytotoxic concentrations of 98.14, 94.97, 89.94, and 86.27 μg/mL for the THP-1, HEK001, WS1, and HMCB cell lines, respectively. Additionally, *R. urticifolius* extracts exhibited an inhibitory capacity on melanin and tyrosinase production, anti-hyaluronidase activity, and an inhibition of elastase and collagenase. These activities suggest that the species *R. urticifolius* holds therapeutic potential without notable cytotoxic effects, being useful in cosmetic and pharmaceutical applications.

Among the isolated compounds, compound **1** shows remarkable biological properties. It effectively inhibits tyrosinase activity (IC_50_ = 14.19 μM), melanin production (IC_50_ = 22.24 μM), NF-κB activation (IC_50_ between 9.82 and 12.72 μM), and hyaluronidase activity (IC_50_ = 79.71 μM) and demonstrates an inhibition of elastase (IC_50_ = 80.13 μM) and collagenase (IC_50_ = 76.59 μM) in THP-1, HEK001, WS1, and HMCB cells. Additionally, it demonstrates antioxidant activity by promoting Nrf2 activation (EC_50_ between 116 and 385 nM), indicating its therapeutic potential due to its wide range of beneficial effects, from regulating skin pigmentation to protecting against inflammation and oxidative stress.

However, it is important to acknowledge the limitations of this study as it was solely based on in vitro models, limiting the extrapolation of results to more complex biological systems. Furthermore, the lack of pNF-κB and Nrf2 expression analysis constitutes an additional limitation that could impact the full understanding of underlying mechanisms. To address these limitations, future research should include comprehensive in vivo studies that not only validate the in vitro findings but also allow for the assessment of regulation of these important molecular pathways.

For future investigations on the *Rubus urticifolius* species, several research directions are proposed. These include elucidating the molecular mechanisms underlying the observed activities and thoroughly exploring the diversity of compounds within this plant species. Additionally, it is suggested to use compound **1** ((*E*)-2-(3-(3,4-dimethoxyphenyl)acrylamido)-*N*-methylbenzamide) as the main compound for derivative synthesis, which could lead to the development of more effective and safer compounds for diseases related to skin pigmentation, protection against inflammations, and oxidative stress.

## Figures and Tables

**Table 1:** IC_50s_ of the inhibition of NF-κB production, calculated for the samples (extracts and compounds of *R. urticifolius*). ^a^ NF-κB IC_50_ values are the means of three independent assays.

Samples No.	NF-κB Inhibition at 72 h IC_50_ ± SEM (μM) ^a^			
	THP-1	HEK001	WS1	HMCB
AqERu (*)	37.39 ± 0.50	33.53 ± 0.95	31.73 ± 0.32	31.21 ± 0.83
HERu (*)	55.47 ± 0.55	50.54 ± 0.47	46.75 ± 0.91	39.14 ± 0.61
DCMERu (*)	29.10 ± 0.35	28.06 ± 0.31	23.03 ± 0.59	19.86 ± 0.75
Celastrol (*)	3.35 ± 0.09	3.33 ± 0.03	3.29 ± 0.05	3.27 ± 0.03
Compound **1**	12.72 ± 0.34	11.24 ± 0.23	11.02 ± 0.67	9.82 ± 0.65
Compound **2**	37.21 ± 0.72	35.42 ± 0.12	32.66 ± 0.51	30.66 ± 0.98
Compound **3**	58.77 ± 0.86	55.97 ± 0.32	53.21 ± 0.23	51.42 ± 0.13
Celastrol	7.62 ± 0.43	7.57 ± 0.23	7.41 ± 0.75	7.36 ± 0.53

(*) = μg/mL. Celastrol = positive control; AqERu = aqueous extract of *R. urticifolius*; HERu = *n*-hexane extract of *R. urticifolius*; DCMERu = dichloromethane/methanol extract of *R. urticifolius*.

**Table 2:** IC_50s_ of the anti-hyaluronidase activity, calculated for the samples (extracts and compounds of *R. urticifolius*). Values are the means of three independent assays.

Samples No.	Anti-Hyaluronidase Activity	
	IC_50_ (μM)	Hyaluronidase Inhibition (%)
AqERu (*)	111.20 ± 2.97	57.43 ± 1.92
HERu (*)	165.30 ± 2.66	36.71 ± 1.75
DCMERu (*)	71.72 ± 1.65	72.54 ± 1.68
Quercetin (*)	250.03 ± 3.10	95.72 ± 1.88
Compound **1**	79.71 ± 1.59	61.12 ± 1.78
Compound **2**	142.59 ± 2.63	30.45 ± 1.92
Compound **3**	171.97 ± 2.97	16.12 ± 1.34
Quercetin	820.01 ± 6.58	99.99 ± 1.19

(*) = μg/mL. Quercetin = positive control; AqERu = aqueous extract of *R. urticifolius*; HERu = *n*-hexane extract of *R. urticifolius*; DCMERu = dichloromethane/methanol extract of *R. urticifolius*.

**Table 3:** IC_50s_ of the anti-elastase and anti-collagenase activities, calculated for the samples (extracts and compounds of *R. urticifolius*). Values are the means of three independent assays.

Samples No.	Anti-Elastase		Anti-Collagenase	
	IC_50_ (μM)	Elastase Inhibition (%)	IC_50_ (μM)	Collagenase Inhibition (%)
AqERu (*)	57.09 ± 1.92	52.86 ± 1.94	56.42 ± 1.32	52.86 ± 1.22
HERu (*)	102.35 ± 1.85	15.49 ± 1.76	75.96 ± 1.96	36.53 ± 1.68
DCMERu (*)	30.42 ± 1.49	74.88 ± 1.51	11.60 ± 1.69	90.31 ± 1.77
EGCG (*)	114.38± 3.07	94.44 ± 1.30	114.04± 3.07	95.29 ± 2.09
Compound **1**	80.13 ± 1.92	60.76 ± 1.44	76.59 ± 1.31	62.24 ± 1.42
Compound **2**	168.11 ± 3.09	17.68 ± 1.46	152.40 ± 3.73	24.87 ± 1.34
Compound **3**	191.58 ± 3.78	6.19 ± 1.44	187.17 ± 2.61	7.73 ± 1.21
EGCG	250.37 ± 3.25	98.08 ± 1.74	250.54 ± 3.65	98.81 ± 1.42

(*) = μg/mL. EGCG = positive control; AqERu = aqueous extract of *R. urticifolius*; HERu = *n*-hexane extract of *R. urticifolius*; DCMERu = dichloromethane/methanol extract of *R. urticifolius*.

## Data Availability

The datasets and materials used and/or analysed during the current study are available from the corresponding author upon reasonable request.

## Supplementary Materials

The following supporting information can be downloaded at: https://www.mdpi.com/article/10.3390/ph17070937/s1. The spectra (NMR and HRESIMS) for the extract and compounds from *R. urticifolius* tested in this study are provided as supporting information (Figures S1–S19). Figure S1: UV-Visible spectrum of *Rubus urticifolius.* Figure S2: IR spectrum of *Rubus urticifolius*. Figure S3: ^1^H NMR spectrum of AqERu in D_2_O 300 MHz. Figure S4: ^1^H NMR spectrum of HERu in CDCl_3_ 300 MHz. Figure S5: ^1^H NMR spectrum of DCMERu in CDCl_3_ 300 MHz. Figure S6: ^1^H NMR spectrum combination of *Rubus urticifolius* extracts in CDCl_3_ 300 MHz. Expansion 1. Figure S7: ^1^H NMR spectrum combination of *Rubus urticifolius* extracts in CDCl_3_ 300 MHz. Expansion 2. Figure S8: ^1^H NMR spectrum of (*E*)-2-(3-(3,4-dimethoxyphenyl)acrylamido)-*N*-methylbenzamide (**1**) in DMSO-*d_6_* 500 MHz. Figure S9: ^13^C NMR spectrum of (*E*)-2-(3-(3,4-dimethoxyphenyl)acrylamido)-*N*-methylbenzamide (**1**) in DMSO-*d_6_* 126 MHz. Figure S10: IR spectrum of compound **2**. Figure S11: ^1^H NMR spectrum of 4-Hydroxyquinoline-2-carboxylic acid (**2**) in CD_3_OD 500 MHz. Figure S12: ^13^C NMR spectrum of 4-Hydroxyquinoline-2-carboxylic acid (**2**) in CD_3_OD 126 MHz. Figure S13: ^1^H-^1^H COSY spectrum of 4-Hydroxyquinoline-2-carboxylic acid (**2**) in CD_3_OD 500 MHz. Figure S14: HREIMS spectrum of 4-Hydroxyquinoline-2-carboxylic acid (**2**). Figure S15: IR spectrum of compound **3**. Figure S16: ^1^H NMR spectrum of (*E*)-2-Cyano-3-(4-hydroxyphenyl)acrylic acid (**3**) in CD_3_OD 500 MHz. Figure S17: ^13^C NMR spectrum of (*E*)-2-Cyano-3-(4-hydroxyphenyl)acrylic acid (**3**) in CD_3_OD 126 MHz. Figure S18: ^1^H-^1^H COSY spectrum of (*E*)-2-Cyano-3-(4-hydroxyphenyl)acrylic acid (**3**) in CD_3_OD 500 MHz. Figure S19: HREIMS spectrum of (*E*)-2-Cyano-3-(4-hydroxyphenyl)acrylic acid (**3**).
