# Supplementary material for: Rubus urticifolius Compounds with Antioxidant Activity, and Inhibition Potential against Tyrosinase, Melanin, Hyaluronidase, Elastase, and Collagenase
Source: Pharmaceuticals (Basel). 2024 Jul 13;17(7):937. doi: 10.3390/ph17070937 (PMC11280343; doi:10.3390/ph17070937)
Supplement: Supplementary file 1 [file pharmaceuticals-17-00937-s001.zip › pharmaceuticals-3112475-supplementary.pdf]

Supplementary Materials

Rubus urticifolius Compounds with Antioxidant Activity, and Inhibition Potential against Tyrosinase, Melanin, Hyaluronidase, Elastase, and Collagenase

Luis Apaza Ticona [1,2,*], Javier Sánchez Sánchez-Corral [2], Carolina Díaz-Guerra Martín [1], Sara Calderón Jiménez [1], Alejandra López González [1] and Cristina Thiebaut Estrada [3]

[1] Organic Chemistry Unit, Department of Chemistry in Pharmaceutical Sciences, Faculty of Pharmacy, University Complutense of Madrid, Plza. Ramón y Cajal s/n, 28040 Madrid, Spain; cardiazg@ucm.es (C.D.-G.M.); sacalder@ucm.es (S.C.J.); alelop23@ucm.es (A.L.G.)

[2] Department of Organic Chemistry, Faculty of Sciences, University Autónoma of Madrid, Cantoblanco, 28049 Madrid, Spain; jssc1998@gmail.com

[3] COBIOSA, Road of Alpedrete, 6, 28400 Collado Villalba, Spain; cristina.thiebaut@cobiosa.com

* Correspondence: lnapaza@ucm.es

Contents:

- **Figure S1**. UV-Visible spectrum of *Rubus urticifolius.*
- **Figure S2**. IR spectrum of *Rubus urticifolius*.
- **Figure S3**. ^{1}H NMR spectrum of AqERu in D_2O 300 MHz.
- **Figure S4**. ^{1}H NMR spectrum of HERu in $CDCl_3$ 300 MHz.
- **Figure S5**. ^{1}H NMR spectrum of DCMERu in $CDCl_3$ 300 MHz.
- **Figure S6**. ^{1}H NMR spectrum combination of *Rubus urticifolius* extracts in $CDCl_3$ 300 MHz. Expansion 1.
- **Figure S7**. ^{1}H NMR spectrum combination of *Rubus urticifolius* extracts in $CDCl_3$ 300 MHz. Expansion 2.
- **Figure S8**. ^{1}H NMR spectrum of (*E*)-2-(3-(3,4-dimethoxyphenyl)acrylamido)-*N*-methylbenzamide (**1**) in DMSO-d_6 500 MHz.
- **Figure S9**. ^{13}C NMR spectrum of (*E*)-2-(3-(3,4-dimethoxyphenyl)acrylamido)-*N*-methylbenzamide (**1**) in DMSO-d_6 126 MHz.
- **Figure S10**. IR spectrum of compound **2**.
- **Figure S11**. ^{1}H NMR spectrum of 4-Hydroxyquinoline-2-carboxylic acid (**2**) in CD_3OD 500 MHz.
- **Figure S12**. ^{13}C NMR spectrum of 4-Hydroxyquinoline-2-carboxylic acid (**2**) in CD_3OD 126 MHz.
- **Figure S13**. ^{1}H-^{1}H COSY spectrum of 4-Hydroxyquinoline-2-carboxylic acid (**2**) in CD_3OD 500 MHz.
- **Figure S14**. HREIMS spectrum of 4-Hydroxyquinoline-2-carboxylic acid (**2**).
- **Figure S15**. IR spectrum of compound **3**.
- **Figure S16**. ^{1}H NMR spectrum of (*E*)-2-Cyano-3-(4-hydroxyphenyl)acrylic acid (**3**) in CD_3OD 500 MHz.
- **Figure S17**. ^{13}C NMR spectrum of (*E*)-2-Cyano-3-(4-hydroxyphenyl)acrylic acid (**3**) in CD_3OD 126 MHz.
- **Figure S18**. ^{1}H-^{1}H COSY spectrum of (*E*)-2-Cyano-3-(4-hydroxyphenyl)acrylic acid (**3**) in CD_3OD 500 MHz.
- **Figure S19**. HREIMS spectrum of (*E*)-2-Cyano-3-(4-hydroxyphenyl)acrylic acid (**3**).

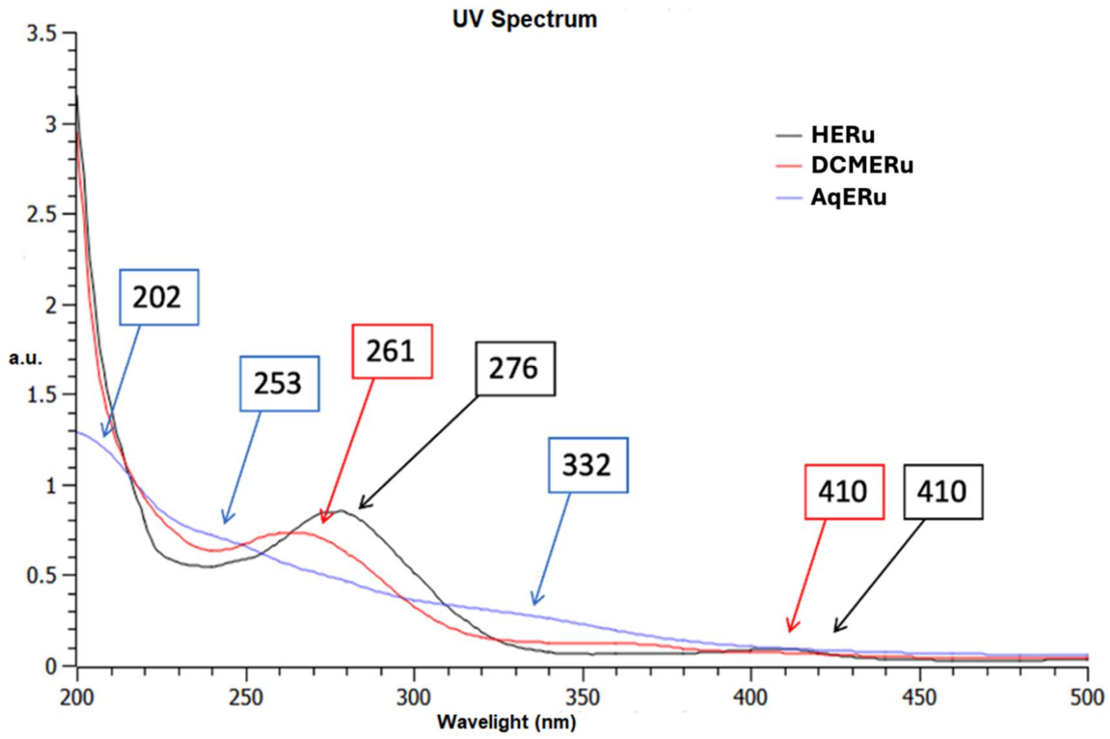

- **Figure S1**. UV-Visible spectrum of *Rubus urticifolius*.

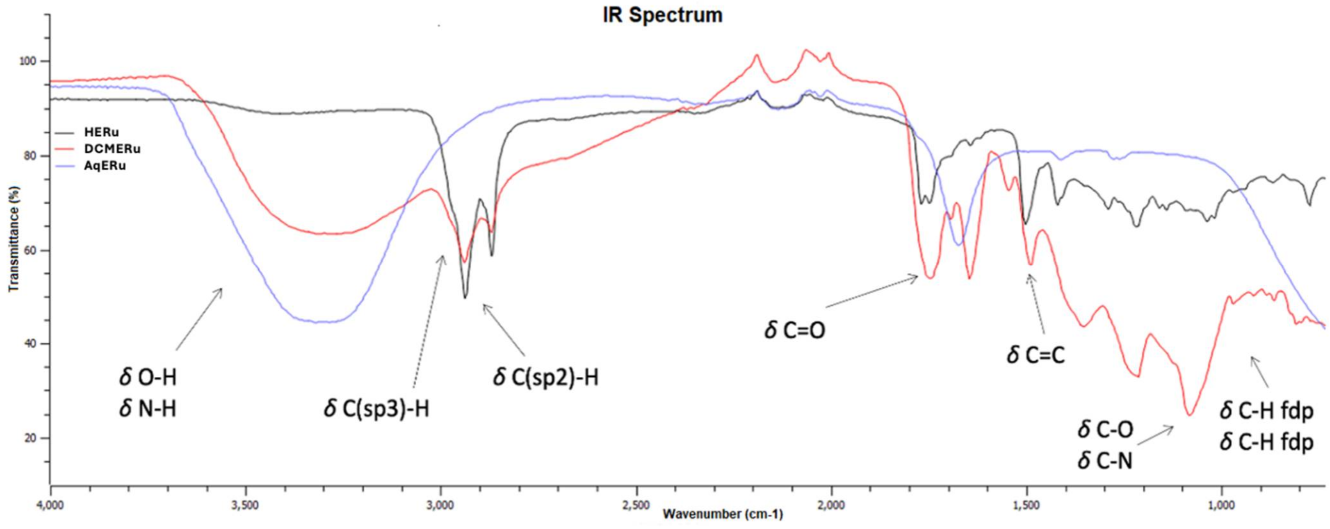

- **Figure S2**. IR spectrum of *Rubus urticifolius*.

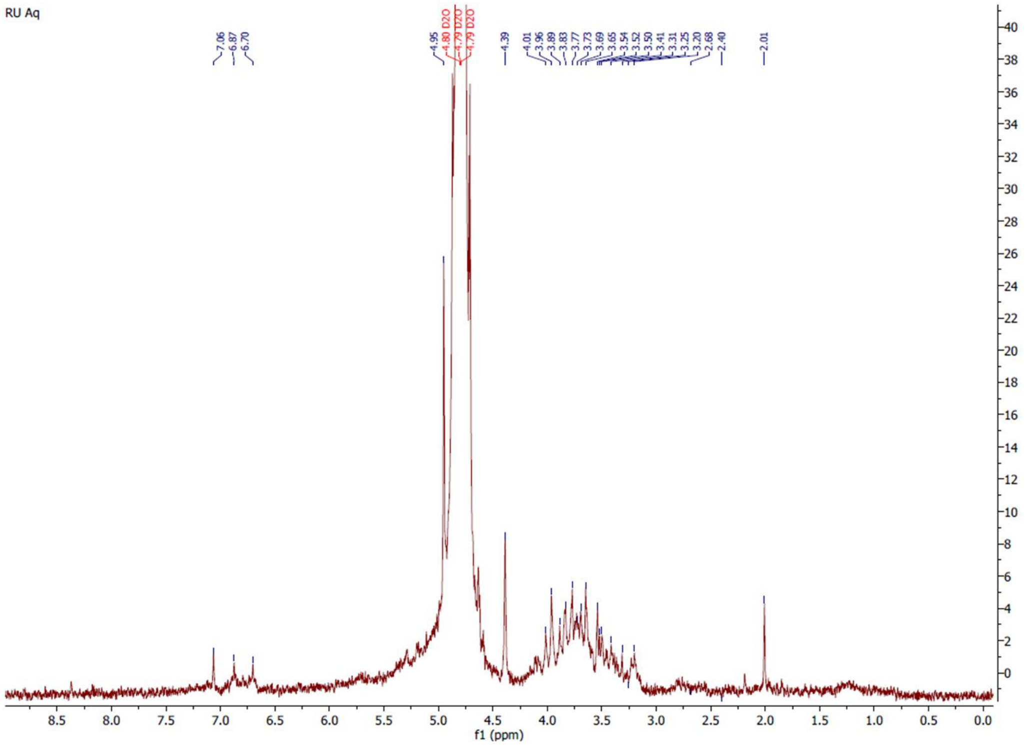

- **Figure S3**. ^{1}H NMR spectrum of AqERu in D_2O 300 MHz.

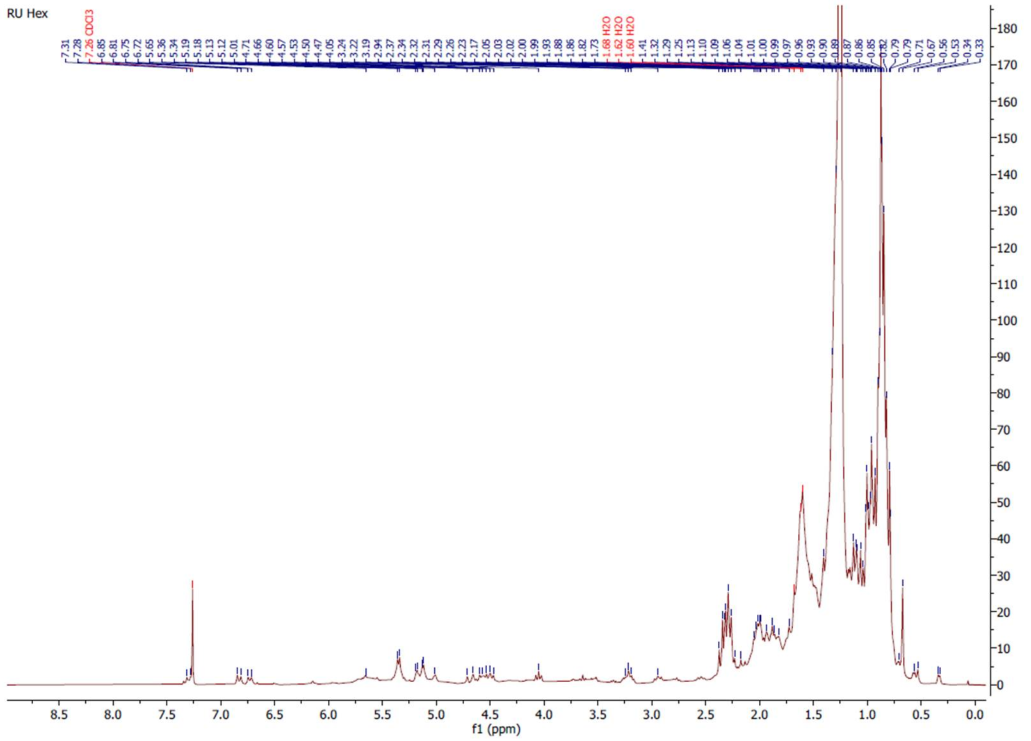

- **Figure S4**. ^{1}H NMR spectrum of HERu in $CDCl_3$ 300 MHz.

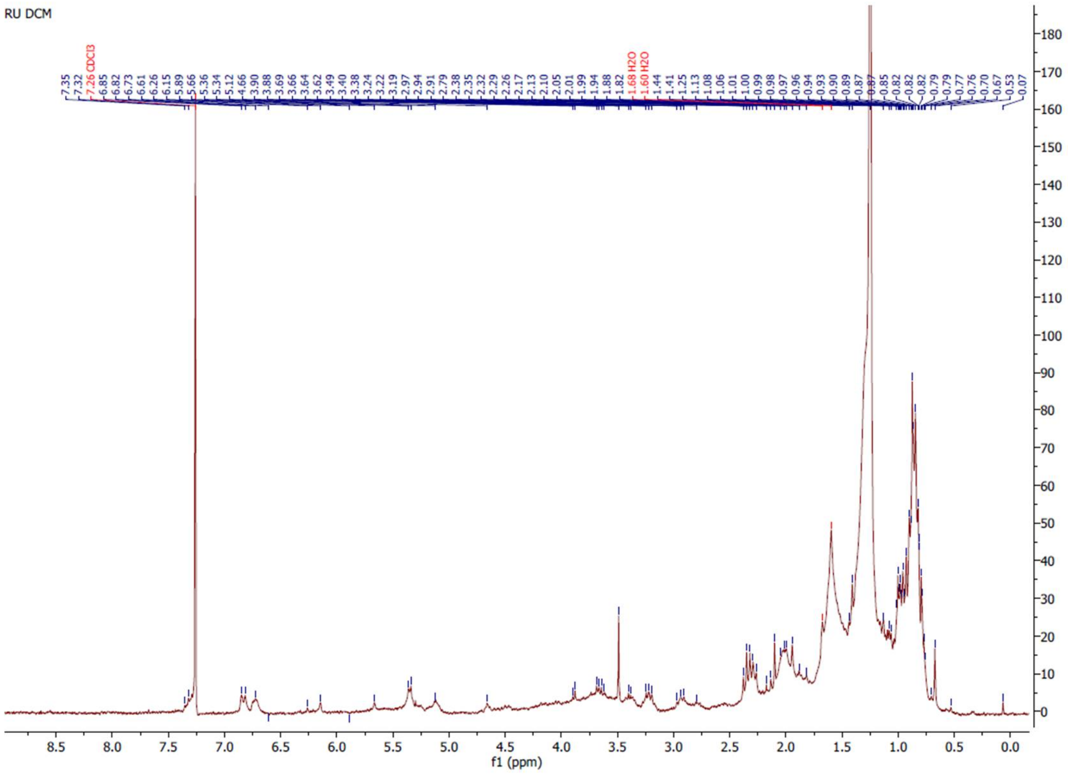

- **Figure S5**. ^{1}H NMR spectrum of DCMERu in $CDCl_3$ 300 MHz.

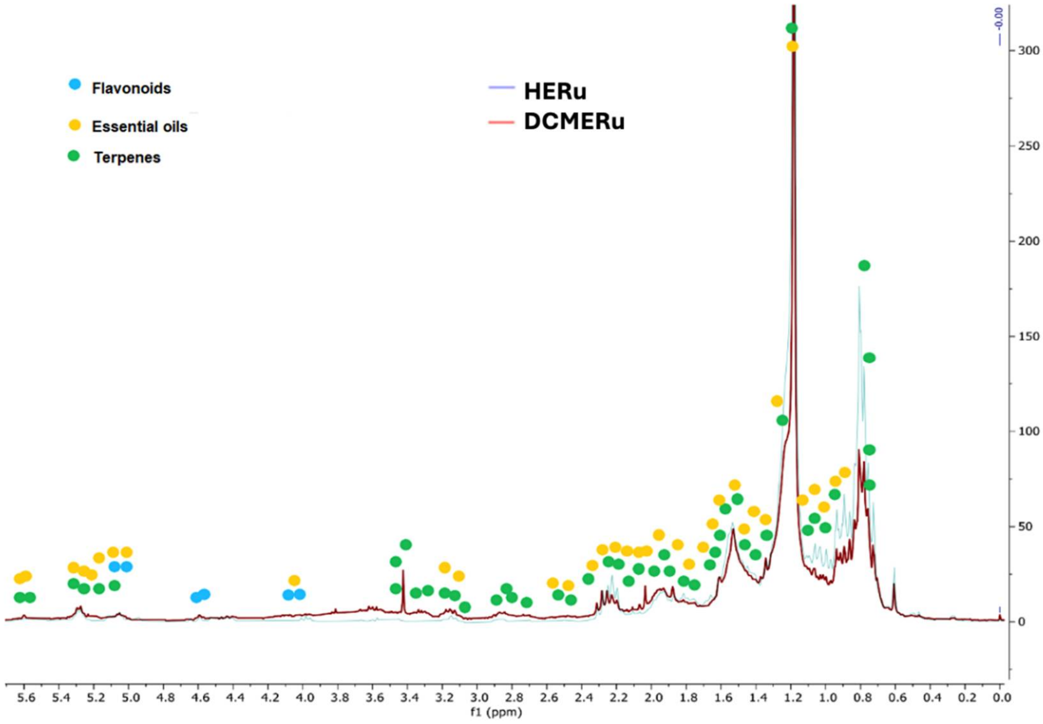

- **Figure S6**. ^{1}H NMR spectrum combination of *Rubus urticifolius* extracts in $CDCl_3$ 300 MHz. Expansion 1.

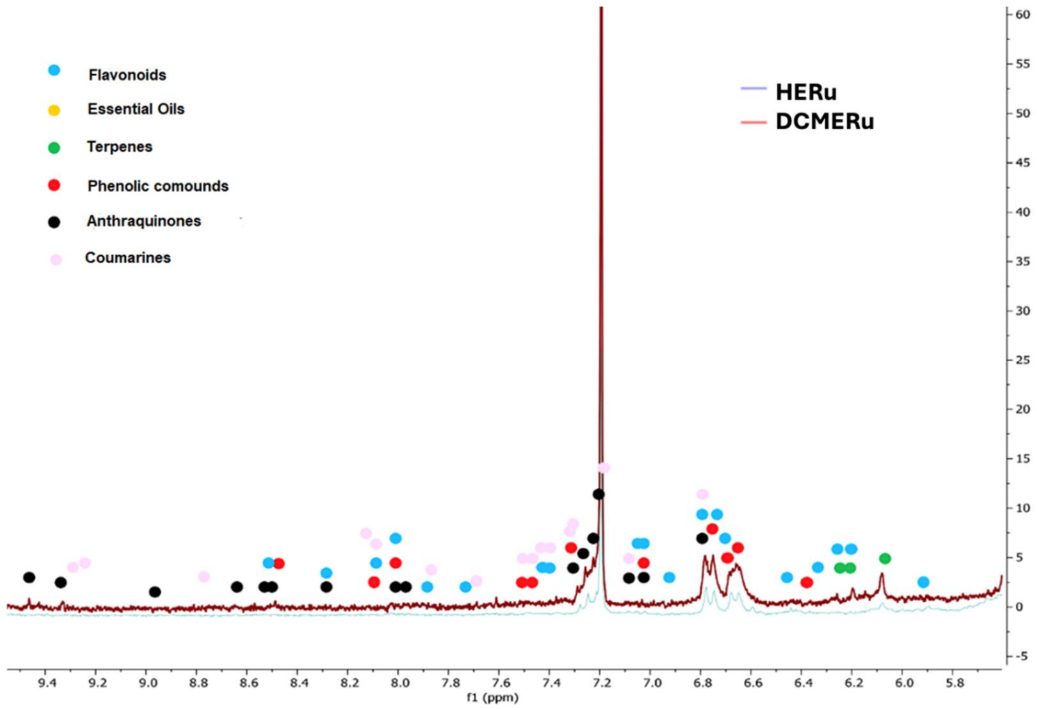

- **Figure S7**. ^{1}H NMR spectrum combination of *Rubus urticifolius* extracts in $CDCl_3$ 300 MHz. Expansion 2.

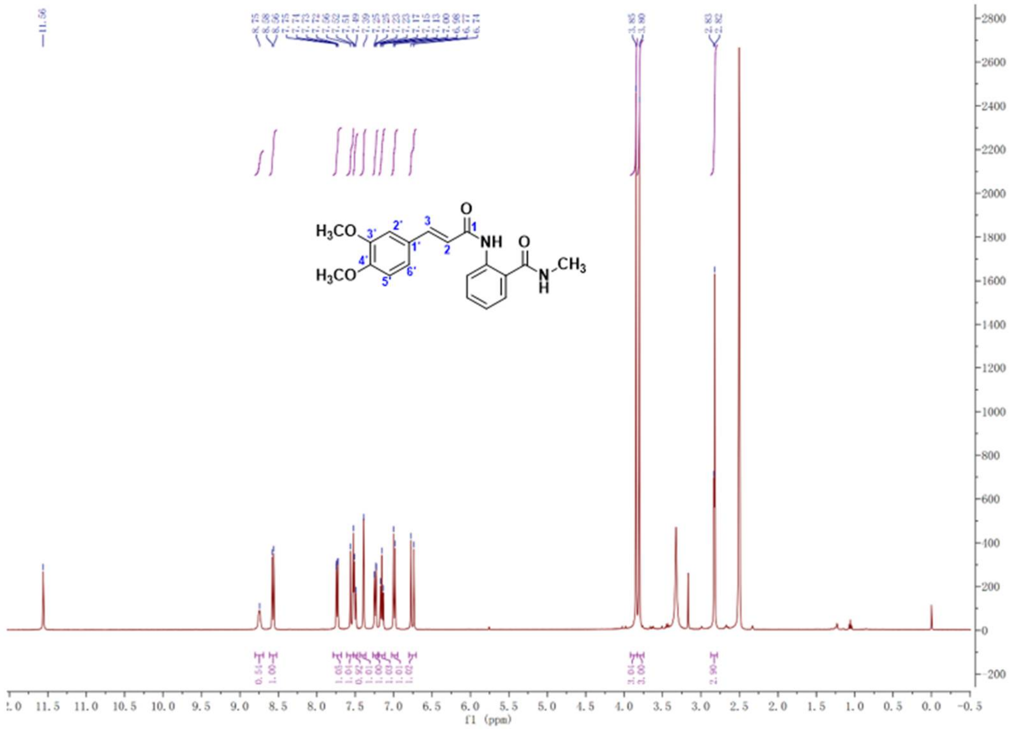

- **Figure S8**. ^{1}H NMR spectrum of (*E*)-2-(3-(3,4-dimethoxyphenyl)acrylamido)-*N*-methylbenzamide (**1**) in DMSO-d_6 500 MHz.

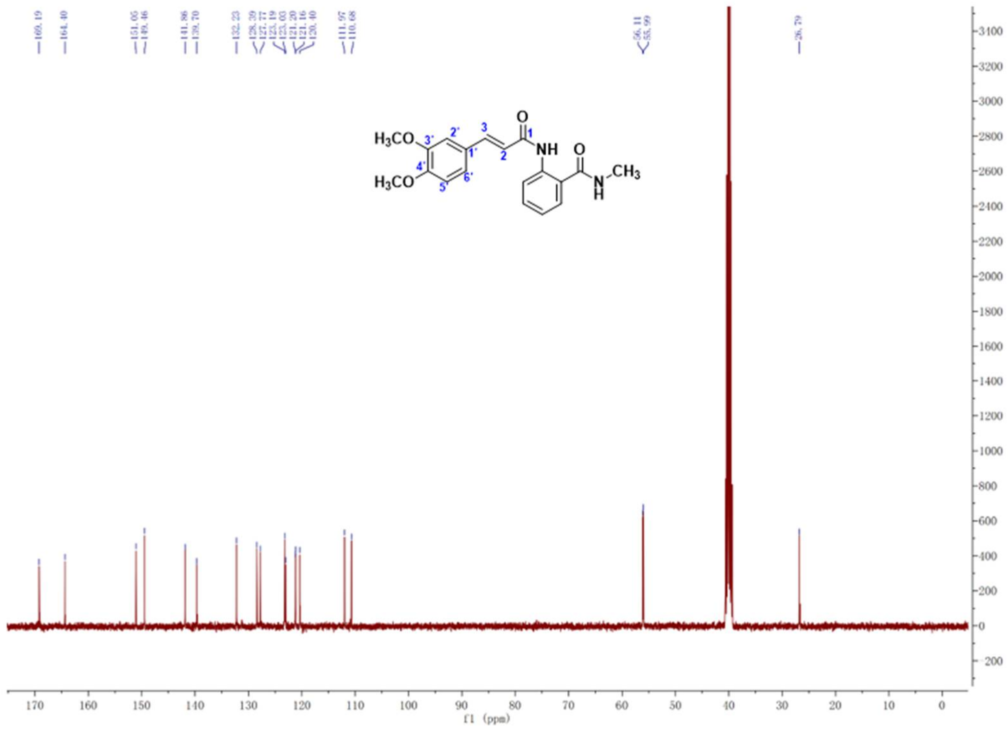

- **Figure S9**. ^{13}C NMR spectrum of (*E*)-2-(3-(3,4-dimethoxyphenyl)acrylamido)-*N*-methylbenzamide (**1**) in DMSO-d_6 126 MHz.

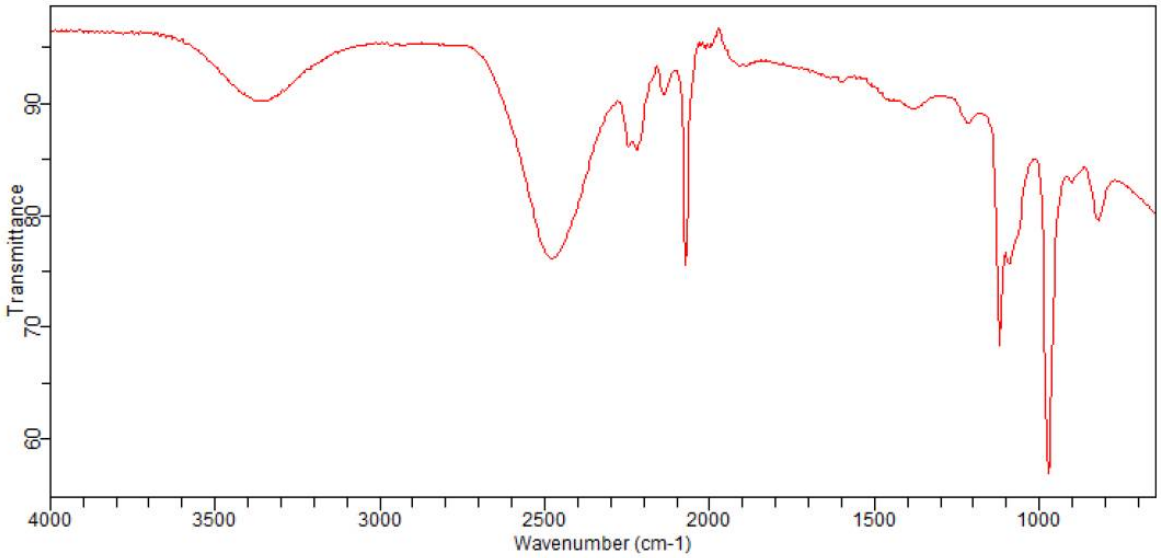

- **Figure S10**. IR spectrum of compound **2**.

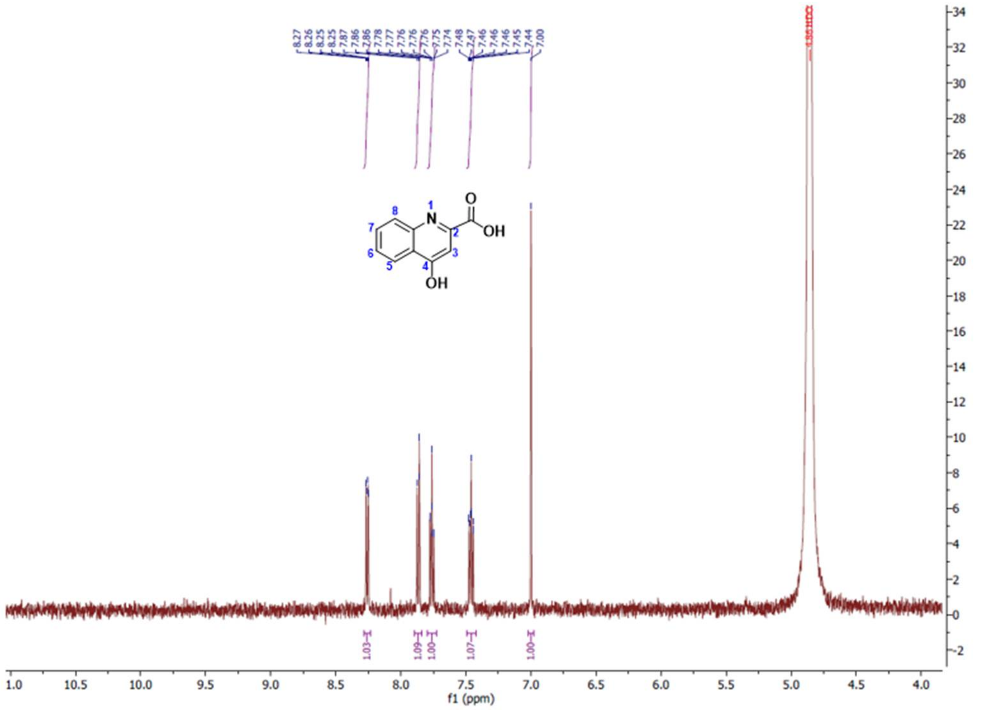

- **Figure S11**. ^{1}H NMR spectrum of 4-Hydroxyquinoline-2-carboxylic acid (**2**) in CD_3OD 500 MHz.

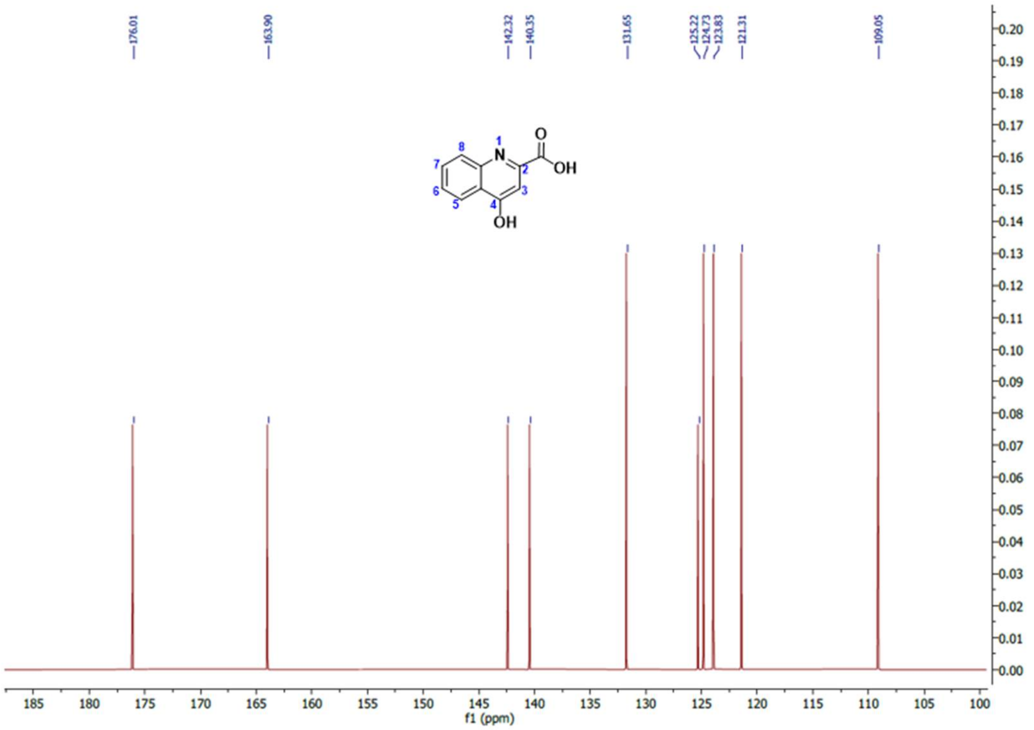

- **Figure S12**. ^{13}C NMR spectrum of 4-Hydroxyquinoline-2-carboxylic acid (**2**) in CD_3OD 126 MHz.

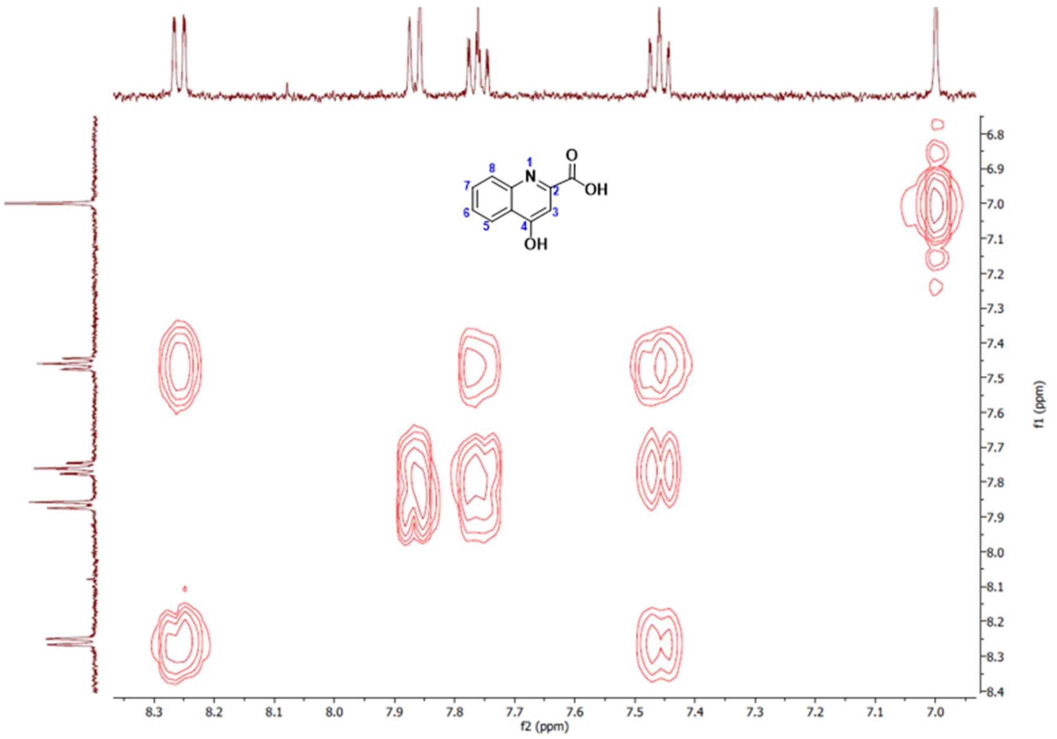

- **Figure S13**. ^1H-^1H COSY spectrum of 4-Hydroxyquinoline-2-carboxylic acid (**2**) in CD_3OD 500 MHz.

Mass Spectrum List Report

Analysis Info

Analysis Name	D:\Data\2022\SS22-219\FS6_LA_RB_2_12887.d	Acquisition Date	5/5/2022 1:48:55 PM
Method	infusion directa POS scan.m	Operator	Demo User
Sample Name	FS6_LA_RB_2	Instrument	impact II 1825265.10157
Comment			

Acquisition Parameter

Source Type	ESI	Ion Polarity	Positive	Set Nebulizer	3.0 Bar
Focus	Active	Set Capillary	4500 V	Set Dry Heater	200 °C
Scan Begin	50 m/z	Set End Plate Offset	-500 V	Set Dry Gas	10.0 l/min
Scan End	2000 m/z	Set Charging Voltage	2000 V	Set Divert Valve	Waste
		Set Corona	0 nA	Set APCI Heater	0 °C

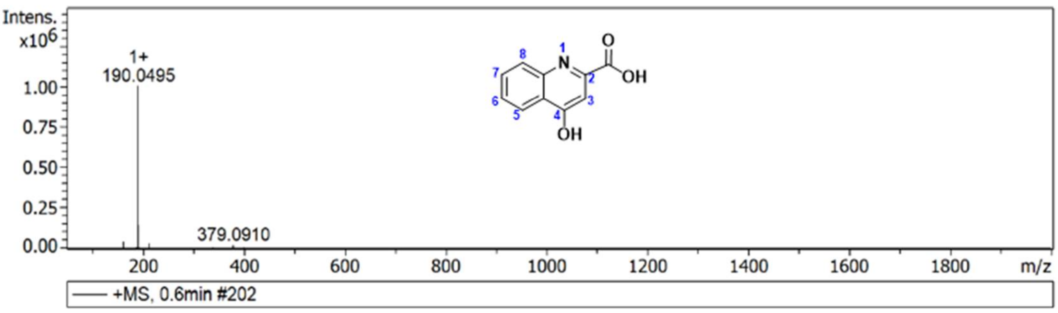

- **Figure S14**. HREIMS spectrum of 4-Hydroxyquinoline-2-carboxylic acid (**2**).

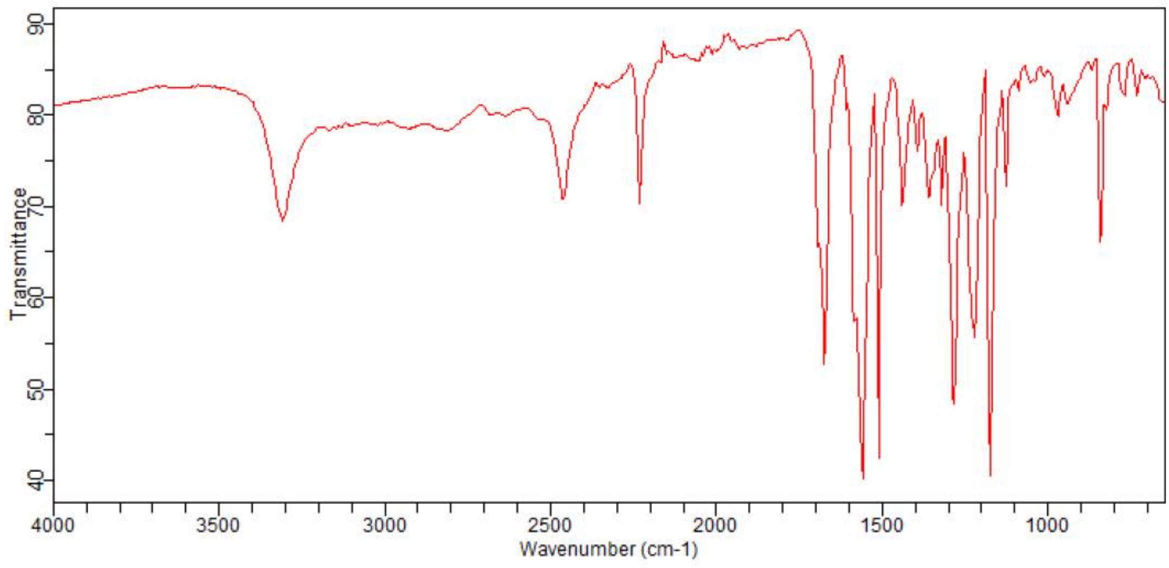

- **Figure S15**. IR spectrum of compound **3**.

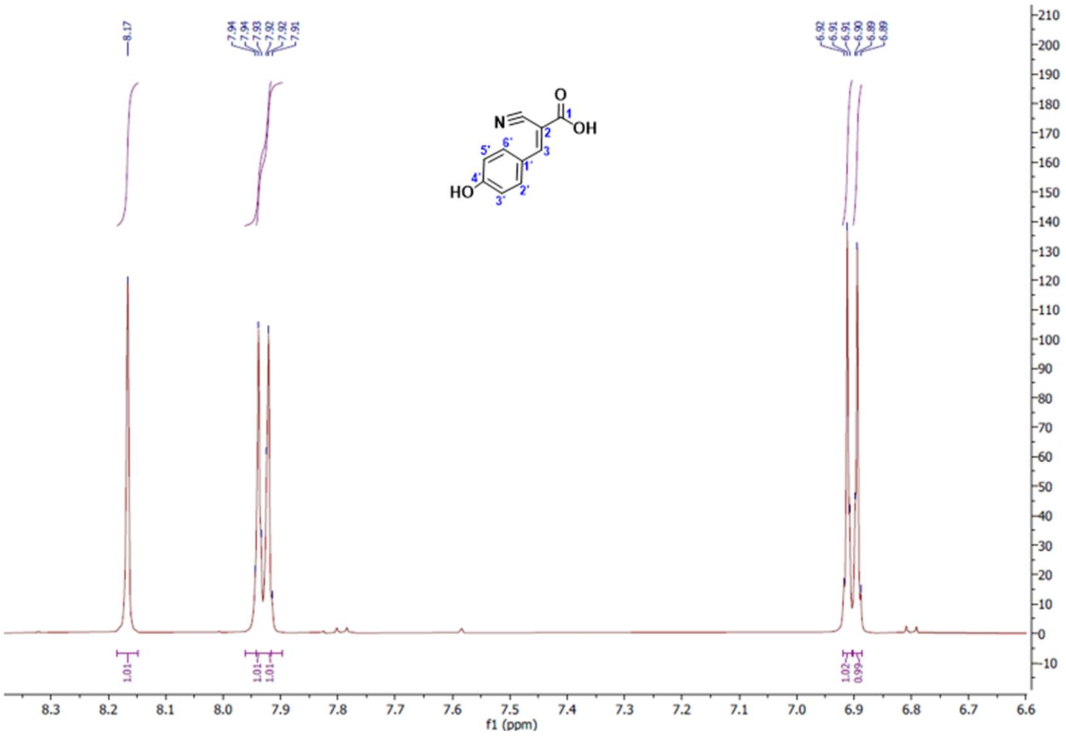

- **Figure S16**. ^{1}H NMR spectrum of (*E*)-2-Cyano-3-(4-hydroxyphenyl)acrylic acid (**3**) in CD_3OD 500 MHz.

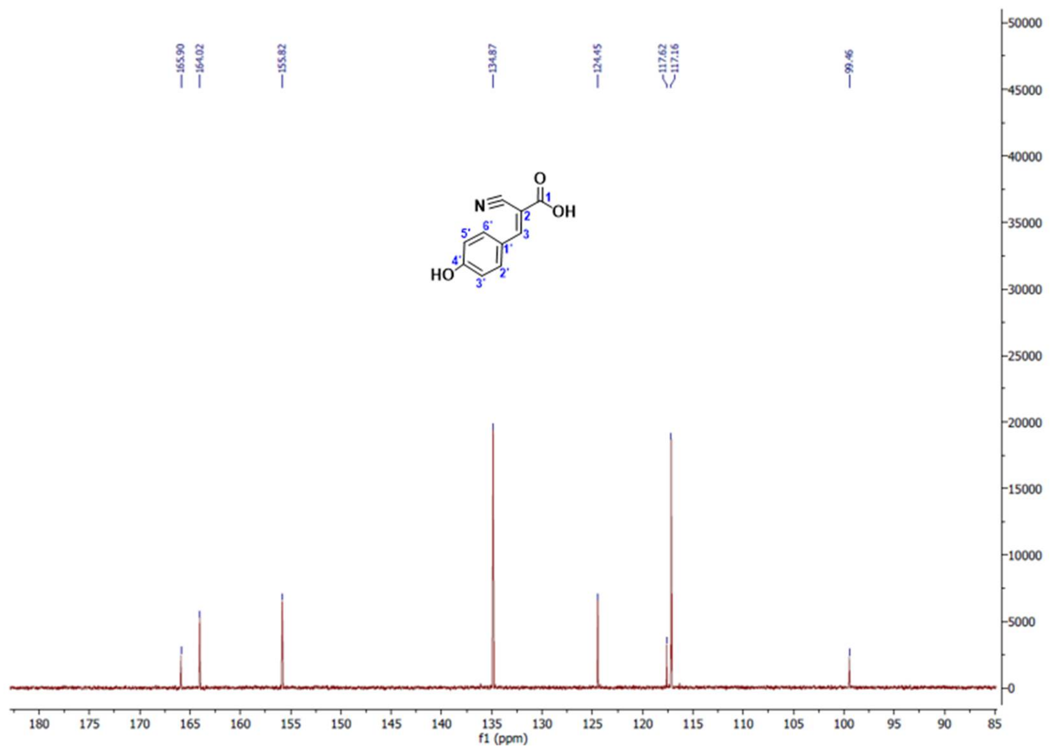

- **Figure S17**. ^{13}C NMR spectrum of (*E*)-2-Cyano-3-(4-hydroxyphenyl)acrylic acid (**3**) in CD_3OD 126 MHz.

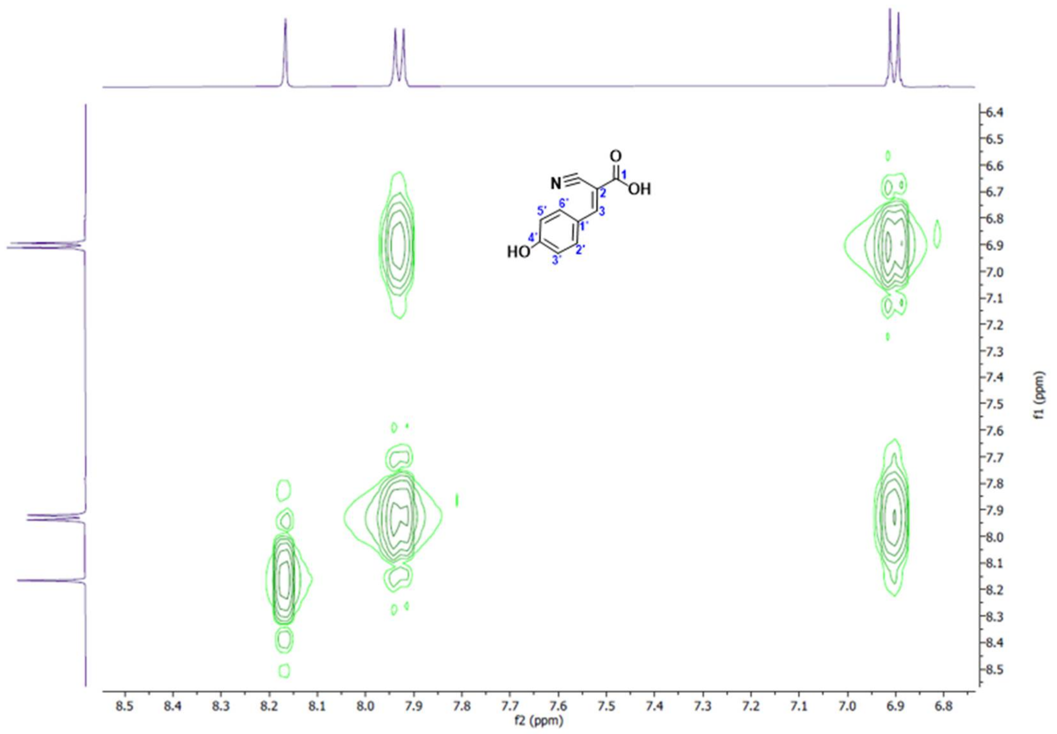

- **Figure S18**. ^{1}H-^{1}H COSY spectrum of (*E*)-2-Cyano-3-(4-hydroxyphenyl)acrylic acid (**3**) in CD_3OD 500 MHz.

Mass Spectrum List Report

Analysis Info

Analysis Name	D:\Data\2022\SS22-219\FS_LA_RB_3_12894.d	Acquisition Date	5/5/2022 3:11:20 PM	
Method	infusion directa POS scan.m	Operator	Demo User	
Sample Name	FS_LA_RB_3	Instrument	impact II	1825265.10157
Comment				

Acquisition Parameter

Source Type	ESI	Ion Polarity	Positive	Set Nebulizer	3.0 Bar
Focus	Active	Set Capillary	4500 V	Set Dry Heater	200 °C
Scan Begin	50 m/z	Set End Plate Offset	-500 V	Set Dry Gas	10.0 l/min
Scan End	2000 m/z	Set Charging Voltage	2000 V	Set Divert Valve	Waste
		Set Corona	0 nA	Set APCI Heater	0 °C

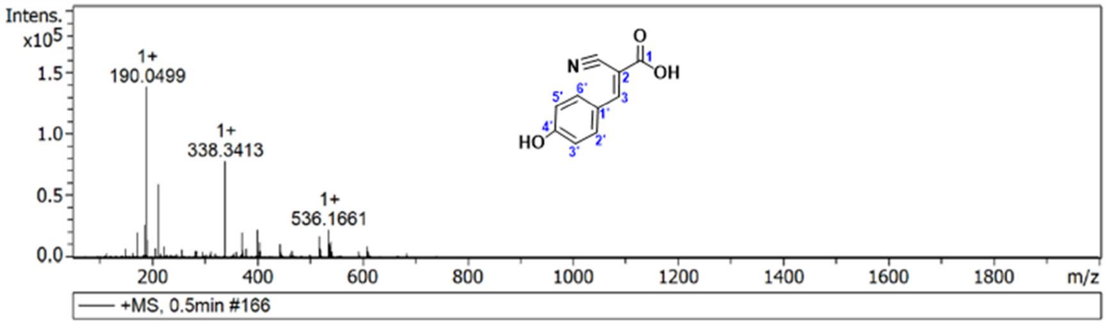

- **Figure S19**. HREIMS spectrum of (*E*)-2-Cyano-3-(4-hydroxyphenyl)acrylic acid (**3**).
